# The stability of educational achievement across school years is largely explained by genetic factors

**DOI:** 10.1038/s41539-018-0030-0

**Published:** 2018-09-04

**Authors:** Kaili Rimfeld, Margherita Malanchini, Eva Krapohl, Laurie J. Hannigan, Philip S. Dale, Robert Plomin

**Affiliations:** 1 Social, Genetic and Developmental Psychiatry Centre, Institute of Psychiatry, Psychology and Neuroscience, King’s College London, London, UK; 2 Department of Psychology, University of Texas at Austin, Austin, USA; 3 Department of Speech and Hearing Sciences, University of New Mexico, Albuquerque, USA

## Abstract

Little is known about the etiology of developmental change and continuity in educational achievement. Here, we study achievement from primary school to the end of compulsory education for 6000 twin pairs in the UK-representative Twins Early Development Study sample. Results showed that educational achievement is highly heritable across school years and across subjects studied at school (twin heritability ~60%; SNP heritability ~30%); achievement is highly stable (phenotypic correlations ~0.70 from ages 7 to 16). Twin analyses, applying simplex and common pathway models, showed that genetic factors accounted for most of this stability (70%), even after controlling for intelligence (60%). Shared environmental factors also contributed to the stability, while change was mostly accounted for by individual-specific environmental factors. Polygenic scores, derived from a genome-wide association analysis of adult years of education, also showed stable effects on school achievement. We conclude that the remarkable stability of achievement is largely driven genetically even after accounting for intelligence.

## Learning: High stability of school achievement

Twin studies have shown that individual differences in school achievement are to a large extent (around 60%) explained by genetic differences. However, little is known about age-to-age stability and change in school achievement. A team led by Kaili Rimfeld used school achievement data from primary school to the end of compulsory education for 6000 twin pairs in the UK-representative Twins Early Development Study sample. Results showed that school achievement is highly heritable across the school years and across subjects studied at school, that school achievement is highly stable, and that this stability is largely explained by genetic factors. The finding of genetically driven stability of school achievement should provide additional motivation to identify children in need of interventions as early as possible, as the problems are likely to remain throughout the school years.

## Introduction

Educational achievement is important to society and to children as individuals. In fact, educational achievement has been shown to be a good predictor of many life outcomes, such as occupational status, happiness, health, and even life expectancy.^1–5^ Influences on educational achievement, including genetic and environmental etiologies, can best be studied during the period of compulsory education when the full range of family characteristics is represented. Compulsory education in the UK culminates with standardized nation-wide exams, the General Certificate of Secondary Education (GCSE). GCSE grades are a gateway to further education, university acceptance, and even later employment, shaping individuals’ life-long educational and professional trajectories. Previous twin research has shown that GCSE performance is highly heritable, and to a lesser extent explained by environmental factors.^6^ However, little is known about whether the same or different genetic and environmental effects contribute to individual differences in achievement over the course of compulsory education. In the present paper, quantitative (twin) and molecular genetic (DNA) methods are used to examine the etiology and developmental course of educational achievement during the primary and secondary education period, culminating in GCSE grades.

There is now converging evidence for the heritability of educational achievement across school years using family designs, such as twin and adoption studies, and DNA-based methods. Twin studies have shown that around 60% of individual differences in school achievement are explained by inherited differences in children’s DNA sequence.^6–16^ This holds when considering overall achievement scores as well as separate school subjects, from Sciences to Humanities.^11,12^ It is also possible to estimate heritability using DNA of unrelated individuals, where small DNA differences between individuals (single-nucleotide polymorphisms (SNPs)) are associated with the individuals’ scores in a trait of interest. Rather than estimating the association between each SNP and the trait, this method estimates the association between the trait and all the SNPs combined.^17,18^ This so-called SNP heritability for educational achievement has been shown to be around 20–30%.^12,19–21^ The SNP heritability is less than that estimated by twin studies partly because SNP heritability is limited to additive effects of common SNPs that are included in current arrays used to genotype SNPs. Because genome-wide association (GWA) studies have the same limitations as SNP heritability, SNP heritability is the current ceiling for the phenotypic variance that GWA studies can explain.

These univariate genetic analyses have shown that the heritability of educational achievement is substantial and consistent across school years, from primary to secondary education and culminating in the GCSEs.^6,9^ However, that conclusion is agnostic about the extent to which the same or different genetic factors contribute to individual differences in educational achievement longitudinally from age to age, that is, to stability and change. Understanding the developmental etiology of educational achievement in this way has considerable potential for illuminating the mechanisms that trigger differences in GCSE performance and, consequently, in educational and professional outcomes.

Multivariate genetic methods can be used to address this question of the etiology of age-to-age stability and change. Using a multivariate twin design we have previously demonstrated that, during the primary school years, genetic and shared environmental factors show substantial stability in English, Mathematics, and Science, while non-shared environmental factors contribute to change.^9^ However, the genetic and environmental etiology of stability and change of educational achievement across the longer span of school years, from primary school to secondary education and beyond, remains unexplored. Only a few longitudinal studies of reading ability have been reported. In one study, the stability of reading, measured as word recognition, was explained largely by genetic factors (around 70%) from primary through secondary school.^22^ Another study found that the etiology of reading fluency across the first five years of schooling, an important developmental time when students transition from ‘learning to read’ to ‘reading to learn’, was characterized by stable genetic and shared environmental influences.^23^ Two additional longitudinal analyses of reading comprehension in two different samples from the UK^24^ and US^25^ also showed substantial genetic stability. However, school achievement involves much more than reading.

To our knowledge, no longitudinal analysis has been conducted to assess the genetic and environmental etiology of continuity and change of educational achievement throughout compulsory education, for specific subjects as well as for general educational achievement. This is the purpose of the current study, which uses longitudinal data from age 7 to 16 on educational achievement from a UK-representative sample of 6000 twin pairs participating in the Twins Early Development Study (TEDS).^26^

We also addressed the issue of stability and change in school achievement, for the first time using DNA-based analyses. In addition to SNP heritability, which was described earlier, another recently developed method predicts academic achievement directly from DNA using specific SNPs that have been shown to be associated with the trait in GWA analyses. This method aggregates thousands of SNP associations, which individually have very small effects, into a genome-wide polygenic score (GPS)^27^ with effects weighted by results from the GWA discovery sample. A GPS can be used to predict variance in a trait for unrelated individuals in a sample independent of the GWA discovery sample. We will refer to this estimate as GPS heritability. It explains less variance than SNP heritability or twin study heritability because GPS heritability predicts educational achievement from specific SNPs.

Our *EduYears* GPS was derived from a GWA study of years of education for 300,000 individuals.^28^ We used the GWA summary data to create an *EduYears* GPS for each of 6000 unrelated individuals (one member of a twin pair) in our TEDS sample ^26^ in the UK. We correlated *EduYears* GPS with achievement measures at ages 7, 9, 12, and 16. We have previously shown that *EduYears* GPS predicts up to 9% of the variance in GCSE scores;^29^ here we extend this analysis and investigate results for specific subjects in addition to general achievement. The focus of our present analyses is the extent to which the *EduYears* GPS contributes to stability of educational achievement.

Genetic stability of school achievement might be explained fully or in part by general cognitive ability (*g*), which has also been shown to be substantially heritable^10,30,31^ and developmentally stable,^32^ and is one of the strongest predictors of school achievement.^33–36^ Moreover, the links between achievement and *g* have shown to be explained by genetic factors.^7,33,37^ Because *g* is a likely candidate to explain stability of school achievement across compulsory education, we also investigate the role of *g* in the stability of educational achievement, using both the twin design and DNA-based methods.

In summary, in this study we use twin analyses and GPS analyses of longitudinal data from TEDS from age 7 to age 16, including GCSE scores, to investigate three issues—the stability of general educational achievement, the stability of achievement in specific subjects, and the contribution of *g* to the stability of educational achievement.

## Results

### Phenotypic analyses

Means and standard deviations were calculated for school achievement across compulsory education for the whole sample, males and females separately, and for all five sex and zygosity groups: monozygotic (MZ) males, dizygotic (DZ) males, MZ females, DZ females, and DZ opposite-sex twin pairs. One twin per pair was randomly selected for phenotypic analyses to maintain independence of data. Analyses of variance (ANOVA) were used to test the significance of these group differences. ANOVA results showed some significant sex differences, however, sex and zygosity together explain only 1% of variance in achievement on average (Supplementary Table 1). For subsequent analyses, the data were corrected for mean sex differences, as described in the Methods section.

### Genetic analyses

#### Univariate genetic analyses

**Fig. 1:**
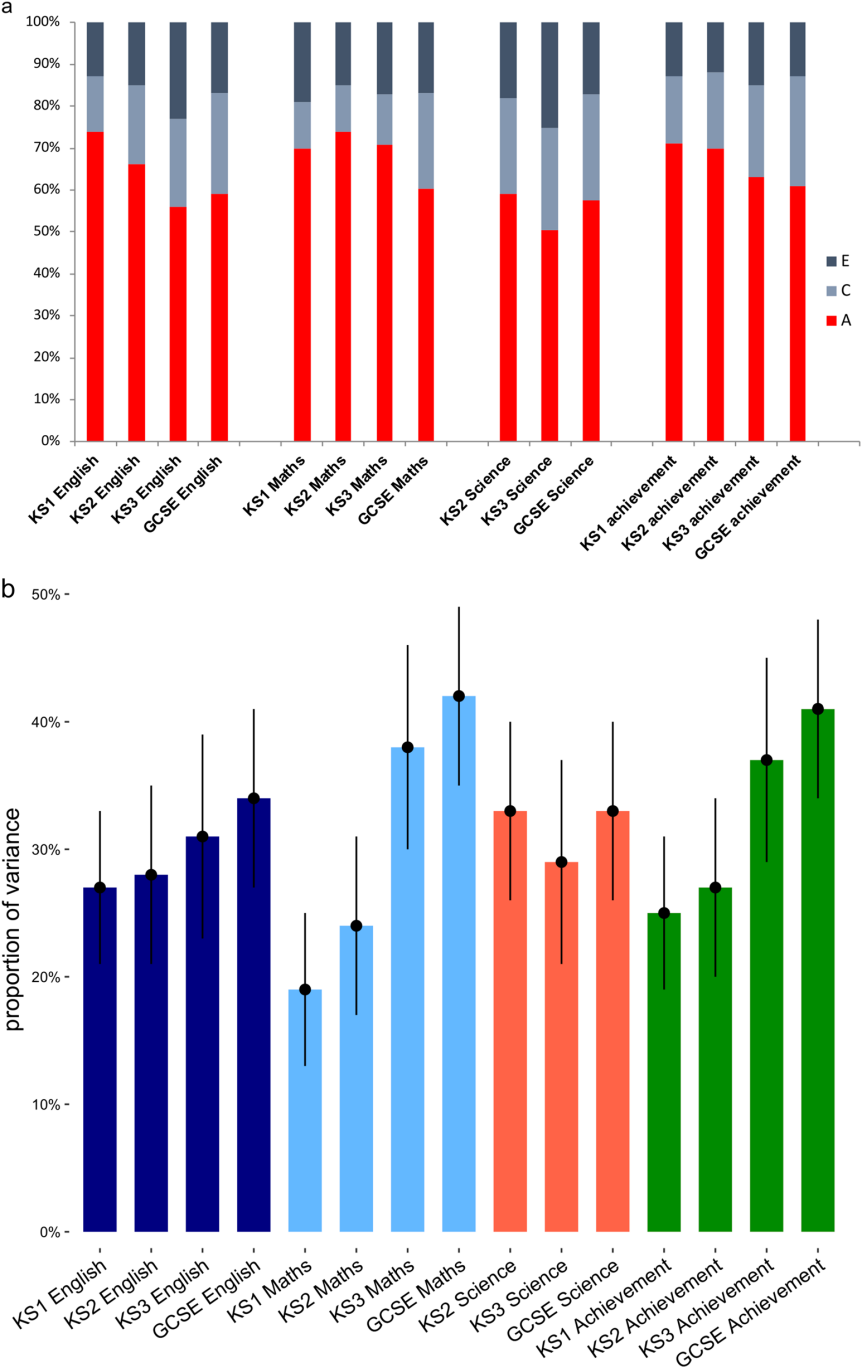
**a** Twin model-fitting results for univariate analyses of educational achievement. A = additive genetic, C = shared environmental, E = non-shared environmental proportions of the variance. **b** SNP heritability estimates of the proportion of variance explained by the additive effects of common SNPs (standard errors as error bars) for the same measures of educational achievement. SNP heritabilities were calculated following adjustment for sex and population stratification. Note: KS1 age 7; KS2 age 11; KS3 age 14; GCSE age 16; Note: Achievement is a composite score of English and Mathematics

Figure 1a presents the twin ACE estimates for achievement across development. All achievement measures show substantial heritability (A ~60%). Shared (C) and non-shared (E) environmental factors both explained about 20% of the variance. Estimates did not vary systematically across subjects or school years. Twin intra-class correlations and parameter estimates with confidence intervals are presented in Supplementary Table 2, which shows that parameter estimates were also similar for teacher ratings, exam performance, and achievement scores that combined teacher ratings and exam performance.

SNP heritabilities were calculated for the same achievement measures using the GCTA package (see Methods). Figure 1b shows that SNP heritabilities were substantial (~30%) but, as expected, only about half as large as the twin estimates, although there was a trend towards increasing SNP heritability with age. For example, the SNP heritability of Mathematics achievement (composite of test scores and teacher ratings) was 19% (SE = 0.06) in KS1 and 38% (SE = 0.08) in KS3 and 42% (SE = 0.07) for GCSE. Twin heritabilities and SNP heritabilities did not differ much across age after the variance accounted for by general congitive ability (*g*) was controlled for by means of linear regression (Supplementary Figure 1). The trend towards increasing SNP heritability with age seen in Fig. 1 disappeared when controlling for *g* (Supplementary Figure 1(b)), which shows increasing heritability with age.^38^

#### Multivariate genetic analyses of age-to-age stability

**Fig. 2:**
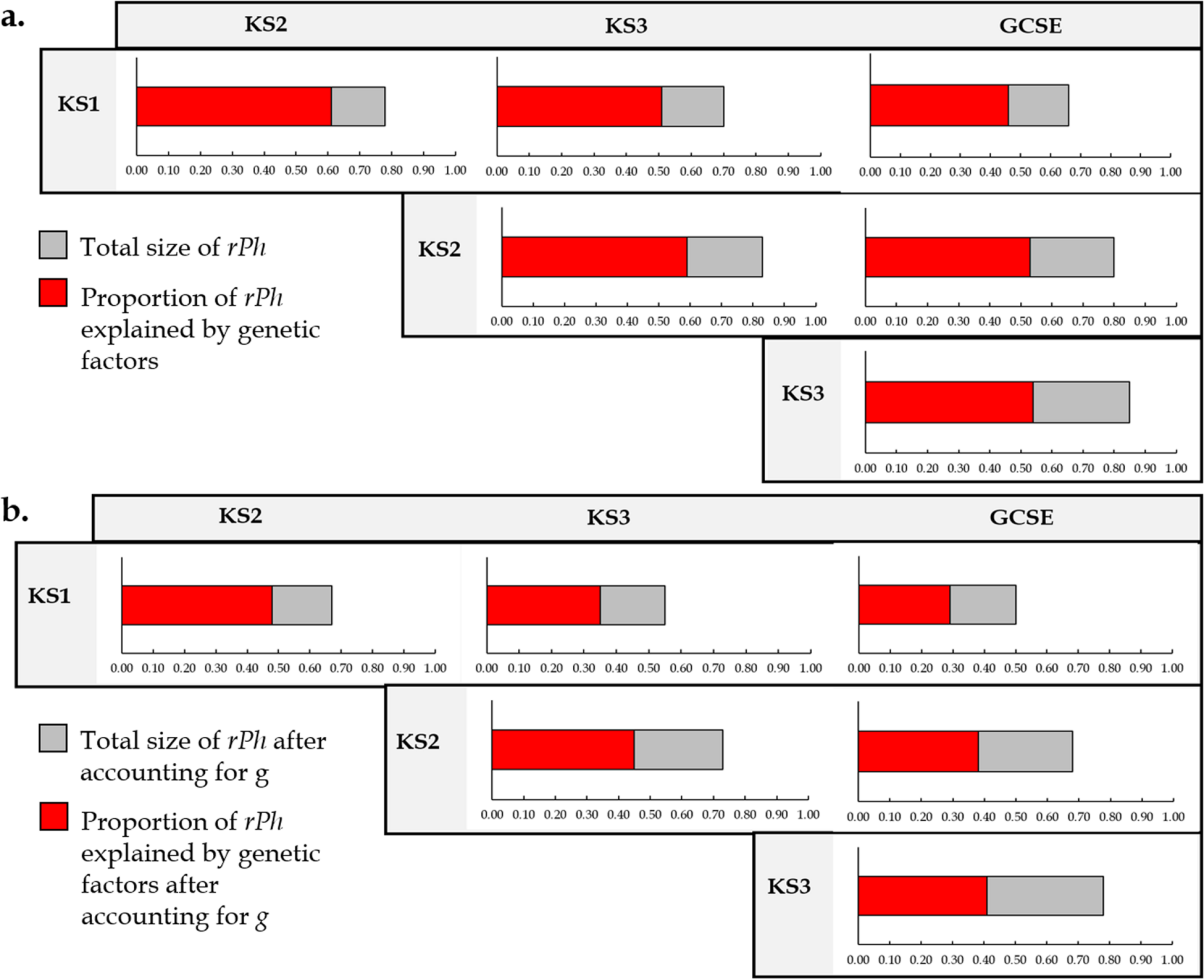
**a** Proportion of the phenotypic correlation (*r*Ph) across ages accounted for by genetic factors**.** **b** Proportion of the phenotypic correlation across ages accounted for by genetic factors after controlling for *g*. Note: KS1 age 7; KS2 age 11; KS3 age 14; GCSE age 16; Note: Achievement is a composite score of English and Mathematics

Academic achievement (measured as the mean of English and Mathematics) was highly stable, with age-to-age correlations ranging from 0.66 to 0.85 (Fig. 2a). In bivariate twin analyses comparing each pair of ages, genetic factors accounted for a substantial proportion of the covariance between ages, explaining from 63 to 79% of the phenotypic correlations (Fig. 2a). Controlling for *g* only slightly reduced the phenotypic stability (range = 0.50–0.78) and genetic stability (range = 0.53–0.82) of the correlations (Fig. 2b). The phenotypic stability from age to age was still mostly accounted for by genetic factors, even after controlling for *g* (52–72%; Fig. 2b). Supplementary Table 3 presents the phenotypic and genetic correlations with 95% confidence intervals for the overall achievement and for separate subjects.

**Fig. 3:**
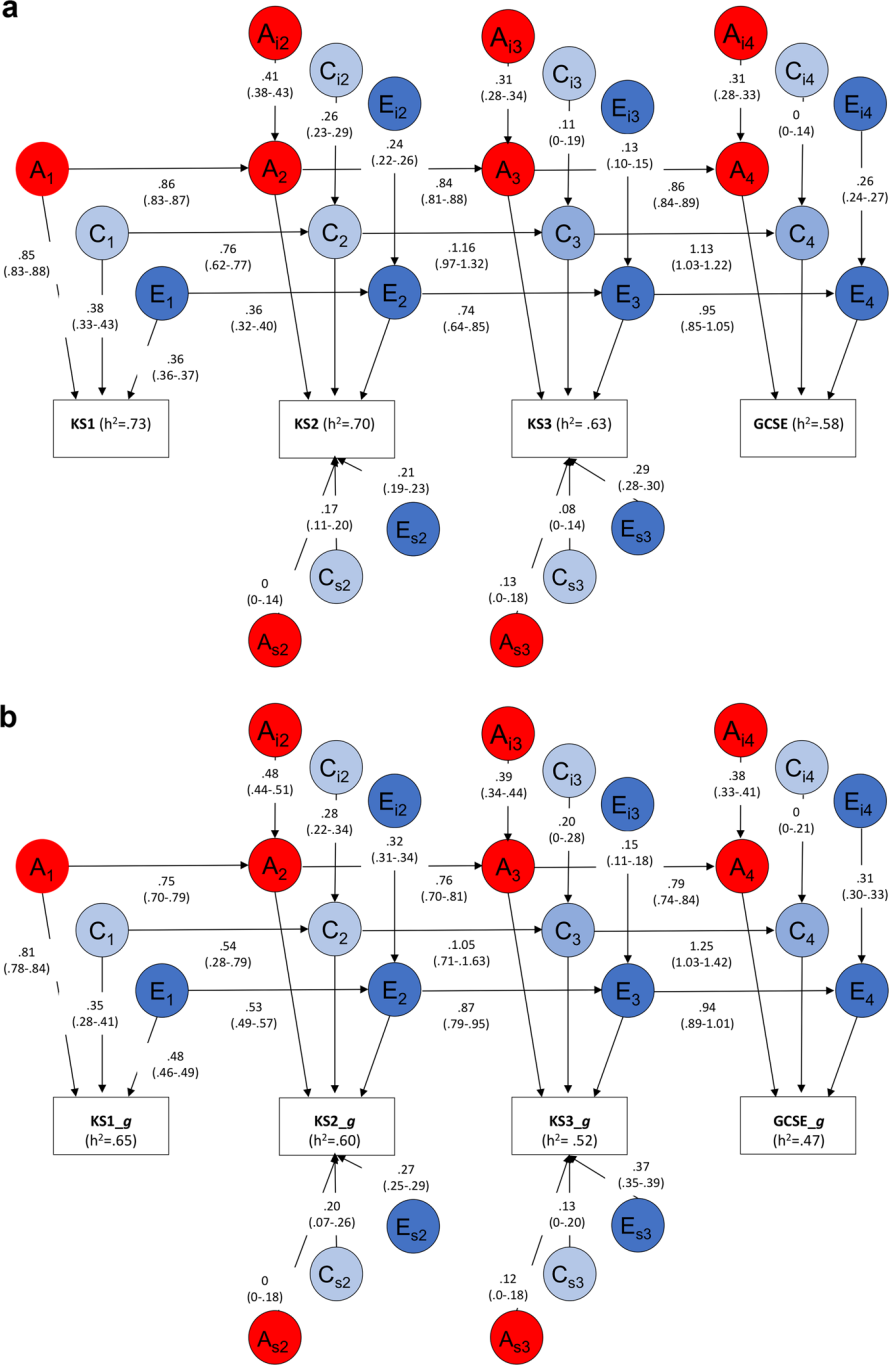
**a** Simplex model-fitting results for stability and change of overall achievement across compulsory education. **b** Simplex model-fitting results for stability and change of overall achievement across compulsory education after controlling for *g*. Note: KS1 age 7; KS2 age 11; KS3 age 14; GCSE age 16; Note: Achievement is a composite score of English and Mathematics, Note: The path estimates are reported rather than standardized variance components

Etiological contributions to stability and change were assessed using multivariate models encompassing all ages of assessment. The first was a simplex longitudinal model^39^ (see Methods and Supplementary Figure 2 for details). The results, presented in Fig. 3, indicate that the stability of core academic achievement was largely explained by additive genetic (A) factors—the genetic paths from age to age are 0.86, 0.84, and 0.86. C was also stable from age to age, accounting for a smaller proportion of variance in academic achievement, amounting to around one-third of the proportion of variance explained by A. E contributed variance that was unique to the measurement occasion, and did not influence subsequent academic achievement across school years, as indicated by the residuals (age-specific effects; E_s2_, E_S3_, and E_i4_). (See Supplementary Figure 3 for the results of simplex model for English, Mathematics and Science separately.)

The proportion of heritability at each age that is accounted for by genetic effects different from those operating at the previous age can be calculated by dividing the sum of the innovation path squared (A_i_) and the age-specific genetic path squared (A_s_) by the overall heritability. For example, for GCSE in Fig. 3a, 17% (i.e., 0.31^2^/0.58) of the heritability of core GCSE performance is innovation (there is no age-specific genetic path); the rest of the heritability (83%) is transmitted from previous achievement ages. For KS3 core achievement, 78% (i.e., 0.70 (heritability of KS2) ×0.84^2^ (genetic transmission)/0.63 (heritability of KS3)) of the genetic variance was transmitted from KS2, and for KS2 77% (0.73 × 0.86^2^/0.70) of the genetic variance was transmitted from KS1. There was substantial innovative genetic influence at each age (A_i_)—24%, 15%, and 17% at ages 12, 14, and 16, respectively. To investigate whether the new genetic influence was due to increasing use of test assessments and decreasing use of teacher assessments across the four ages, we repeated the analyses using only standardized test scores across the school years (Supplementary Figure S4), but the results were highly similar. The remaining genetic variance (0% at age 12 and 3% at age 14) was age specific (path A_s_), in other words, not operating at the previous age and not transmitted to the next age. These paths were not significant as indicated by their 95% confidence intervals.

We also repeated the simplex models statistically controlling for *g* (Fig. 3b). The heritability of core school achievement was somewhat lower after controlling for *g*, comparable to the bivariate genetic results shown in Fig. 2. Nonetheless, educational achievement continued to be highly stable and its stability was still largely accounted for by genetic factors; genetic paths from age to age are 0.75, 0.76, and 0.79.

**Fig. 4:**
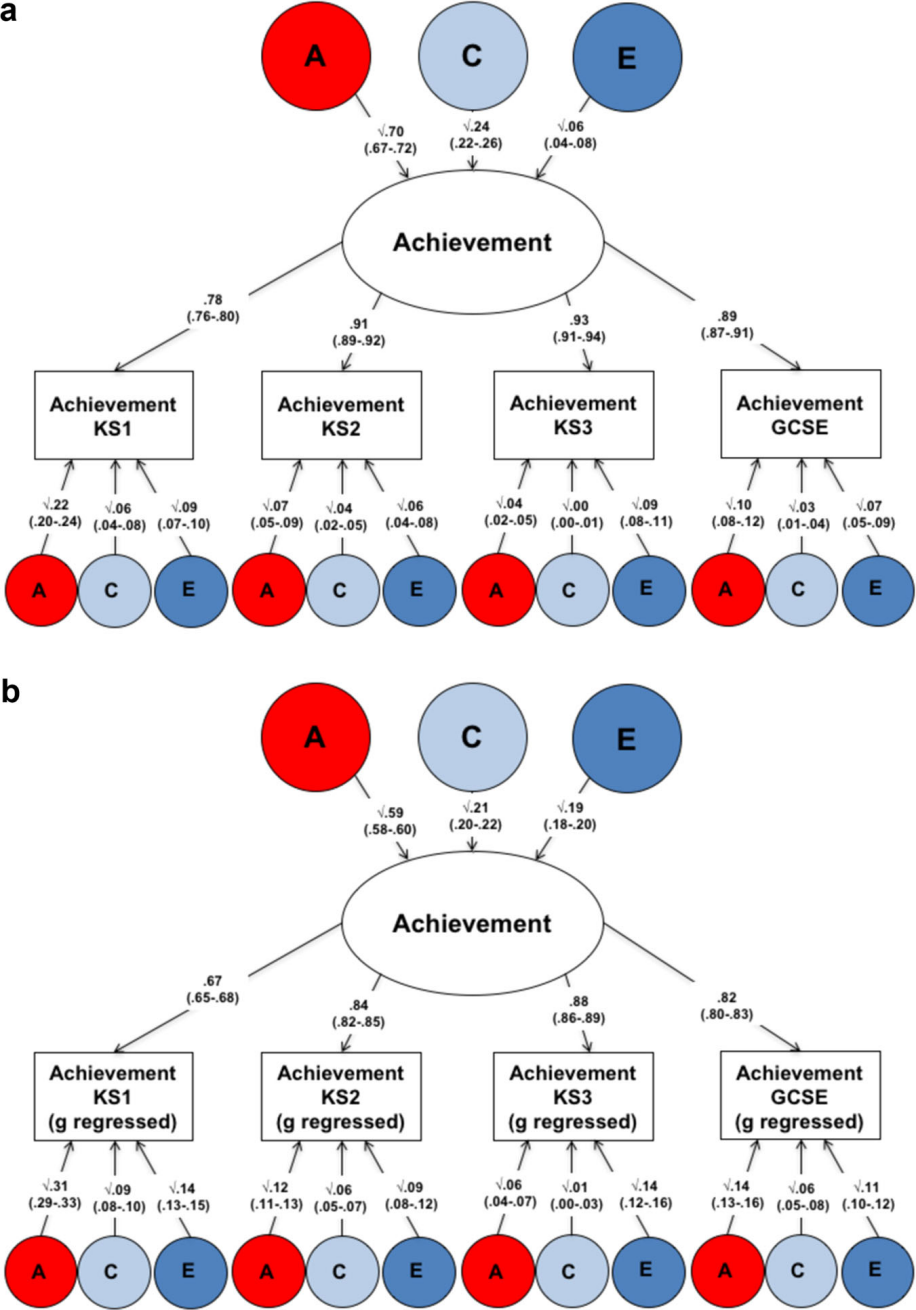
**a** Common pathway model presenting the standardized squared path estimates for overall achievement. **b** and for overall achievement when controlling for *g*. A = additive genetic, C = shared environmental, and E = non-shared environmental components of variance. Note: KS1 age 7; KS2 age 11; KS3 age 14; and GCSE age 16; Note: Achievement is a composite score of English and Mathematics

In order to assess how much variance in the stability of core educational achievement is explained by a single genetic factor, a genetic common pathway model was used (See Methods and Supplementary Figure 5). The results of the common pathway model are presented in Fig. 4. Seventy percent of the overall stability of core educational achievement across compulsory education (heritability of the latent factor) was explained by genetic factors; 24% of the stability of educational achievement was explained by shared environmental factors (Fig. 4a). The results were similar when we controlled for *g—*genetic factors explained 59% of the stability in core educational achievement after controlling for intelligence, 21% of the stability was explained by shared environmental factors (Fig. 4b).

#### GPS analyses

**Fig. 5:**
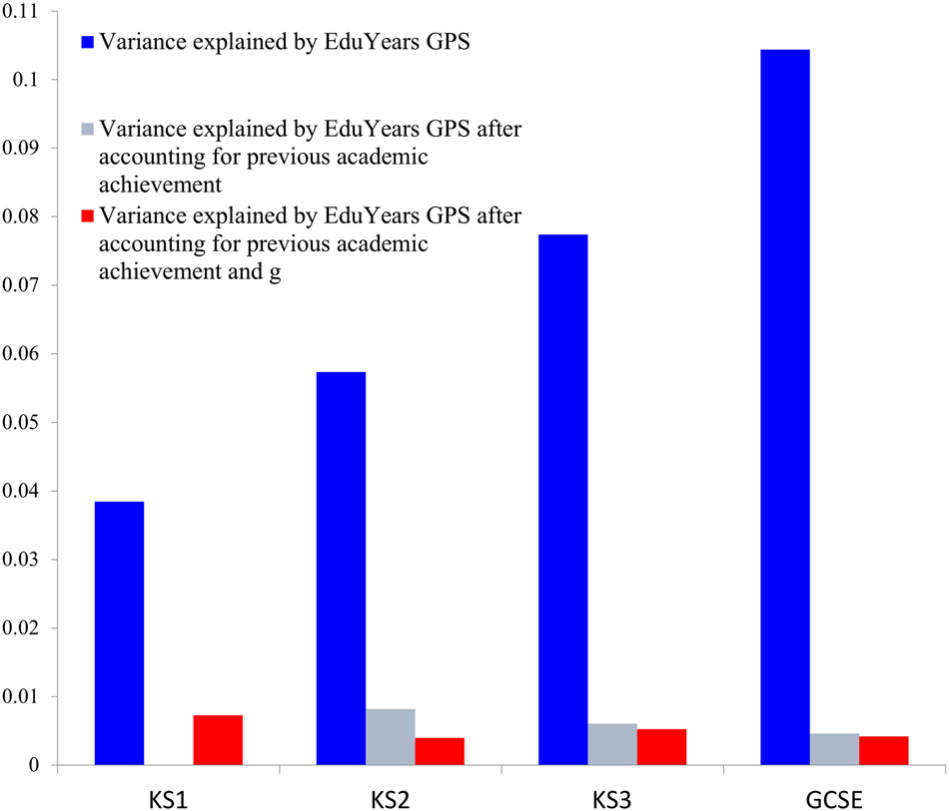
Variance explained by GPS (*EduYears*) using Gaussian mixture weights of 1.0 for overall educational achievement across compulsory education. Note: KS1 age 7; KS2 age 11; KS3 age 14; GCSE age 16; Note: Achievement is a composite score of English and Mathematics

As a complement to our twin results, we investigated genetic stability for core educational achievement using a different methodological approach: *EduYears* GPS. *EduYears* GPS increasingly predicted core educational achievement—about 4% for KS1, 6% for KS2, 8% for KS3, and 10% for GCSE. In order to address the question of genetic stability and innovation, we explored the age-specificity of the *EduYears* GPS prediction, after accounting for the variance explained at all preceding ages. In line with the multivariate twin analyses, *EduYears* GPS’ prediction of educational achievement was largely stable from age to age (Fig. 5). That is, our regression analyses indicated little (<1%) age-specific genetic prediction once the stable prediction of *EduYears* GPS from all previous ages was taken into account. Details of these analyses for core achievement, for subjects separately, and controlling for *g* and previous achievement are presented in Supplementary Table 4. In summary, results were similar for separate subjects and after controlling for *g* and previous achievement. However, *EduYears* GPS still predicts educational achievement when only controlling for *g*, explaining around 4% in GCSE performance, as illustrated in Supplementary Figure 6.

## Discussion

The present study shows that individual differences in educational achievement are highly stable across the years of compulsory schooling from primary through secondary school. Children who do well at the beginning of primary school also tend to do well at the end of compulsory education for much the same reasons. The very high stability of academic achievement across compulsory school years is an interesting finding, particularly when considering that children go through major cognitive and emotional changes from childhood to adolescence, as well as experiencing changes in teachers, friends, and schools.

In addition, the nature of educational achievement also changes during the school years as children are exposed to more subjects and more complex subjects. For reading, children move from learning to read to using reading to learn. The present twin analyses address, for the first time, the etiology of the stability of academic outcomes over compulsory education, showing that genetic factors are largely responsible for this stability. In other words, the same genetic factors largely shape individual differences in achievement from primary through secondary school. Shared environmental factors were also largely stable, although they explained a smaller proportion of overall variance in achievement. However, it has been suggested that shared environmental effects might actually be driven genetically.^40,41^ We show that age-to-age change in achievement scores was to a large extent explained by non-shared environmental factors. This is another example of the general rubric of behavioral genetics, “genetic stability, environmental change”.^22,30,42–44^ We also noted some genetic innovation (change) at each stage of assessment, but, consistent with an overall pattern of stability, all of these new genetic influences were transmitted to the next achievement stage.

A reasonable assumption is that the substantial genetic stability observed here is explained by general cognitive ability (*g*, intelligence). Importantly, however, we showed that the heritability of educational achievement over school years and its stability is not explained by *g* alone. The results of our twin analyses showed that when *g* was controlled for, educational achievement remained highly heritable and stable and the stability of educational achievement independent of *g* was still explained by genetic factors. Although there was evidence for some specific (new) genetic influence at each age, again these new genetic influences were not age specific but were transmitted to the next assessment stage. This is in line with our earlier reports in which we showed that educational achievement at age 16 is not explained by intelligence alone.^10,12^ The *EduYears* GPS regression analysis yielded similar results showing genetic stability, even after controlling for *g*. This GPS result is not exactly analogous to the twin study results, as we tested the effect of the same genetic variants over time. Nevertheless, our multi-method approach yielded similar results indicating that the substantial stability of educational achievement is to a large extent explained by genetic factors.

As new, more powerful GWA studies are conducted, the predictive power of the *EduYears* GPS prediction is likely to increase. The GPS calculated using the 2013 *EduYears* GWA summary statistics with a sample size of 126,000^45^ predicted around 3% of variance in educational achievement in TEDS,^20^ compared to 10% of variance explained in the current study based on the 2016 *EduYears* GWAS with a sample size of 330,000. Another more powerful GWA of educational attainment was recently published, involving over one million participants, this is likely to be a game changer in terms of predictive power.^46^

It should be noted that *EduYears* GPS predicts only about 4% of the variance in adult years of education (educational attainment)^28^ in independent samples, but it predicts more than twice as much variance in GCSE scores at age 16. We are not aware of any other example in which a GPS predicts less variance in the GWA target trait (educational attainment) than in another trait (GCSE scores). We suggest that the reason for this unusual finding is that educational attainment is a much coarser measure than GCSE scores, which are the result of hours of standardized assessment. In support of this hypothesis, we find that *EduYears* GPS also predicts 4% of the variance when we analyzed a similarly coarse dichotomous item about whether or not TEDS participants planned to go to university. Furthermore, *EduYears* GPS also predicts 4% of the variance in a cruder measure of GCSE achievement—5 passes at grades A* to C, which is often used in government statistics, and used for selection purposes by many employers and educational institutions (Supplementary Table 4).

The limitations of this study include the usual assumptions of the twin design, which are described in detail elsewhere.^43,47^ One of these limitations involves assortative mating, in which mate selection is not at random but is instead based on trait similarity. Assortative mating on cognitive abilities and educational achievement has been shown to be substantial (~0.40).^36,43,48^ In the twin design, assortative mating increases DZ correlations relative to MZ correlations and could therefore lead to underestimating heritability and overestimating shared environmental influence; in effect this makes the present findings concerning heritability conservative. GCTA and GPS methods also have their limitations. Notably, both of these DNA-based methods rely on the additive effects of common SNPs genotyped on SNP arrays, and do not capture gene-gene or gene-environment interplay or the effects of less common SNPs.^49^ However, since the main limitations are different for each method used in the current study, the fact that our multi-method approach yielded similar results is a strength.

Our multi-method analyses corroborated previous findings showing that individual differences in educational achievement are largely explained by inherited differences in DNA sequence. The novel contribution of our study is to show that the substantial stability of educational achievement across compulsory education is to a large extent explained by genetic factors, even after controlling for *g*.

Our finding of genetically driven stability of educational achievement should provide additional motivation to identify children in need of interventions as early as possible, as the problems are likely to remain throughout the school years. GPS prediction, specifically, might in the future provide a tool to identify children with educational problems very early in life and aid in providing both individualized prevention and individualized learning programs. We hope that with GPS, we can move towards precision education, just as medicine is moving towards precision medicine.^50,51^ For example, GPS could be used to identify children at birth at genetic risk for developing reading problems, thus enabling early intervention. As preventive interventions have greater chances of succeeding early in life, a great strength of GPS is that they can predict at birth just as well as later in life, which enables early intervention, particularly for those children who are likely to struggle the most.

## Methods

### Participants

The present study used the TEDS sample. TEDS is a large twin study that recruited over 16,000 twin pairs born between 1994 and 1996 in England and Wales. More than 10,000 twin pairs are still actively involved in the study. Rich cognitive and behavioral data, including educational achievement, have been collected from the twins, their parents and teachers, over compulsory education and beyond. Importantly, TEDS was a representative sample of the UK population at first contact, and remains representative in terms of family socioeconomic status and ethinicity.^26,52^ Ethical approval for this study was received from King’s College London Ethics Committee.

The sample for the present study included all twins with available academic achievement measures over the school years. Participants who had major medical or psychiatric conditions, or those with severe perinatal complications, were removed from the analyses. Zygosity was assessed by the parent-reported questionnaire of physical similarity. This measure has been shown to be highly reliable.^53^ Nevertheless, DNA testing was conducted when zygosity was unclear from the questionnaire. The sample size per academic achievement measure is shown in Supplementary Table S1.

DNA has been genotyped for a subsample of unrelated individuals from TEDS (one twin per pair). We processed genotypes for 6710 individuals using the standard quality control procedure followed by imputation of genetic variants to the Haplotype Reference Consortium^54^ (see Supplementary Methods). We then matched the individuals with genotyped data to those participants with available academic achievement data.

### Measures

#### Measures of educational achievement obtained by TEDS

TEDS has obtained assessments of academic achievement directly from the twins’ teachers who reported grades following the UK National Curriculum guidelines, a standardized core academic curriculum formulated by the National Foundation for Educational Research (NFER) and the Qualifications and Curriculum Authority (QCA) (NFER: http://www.nfer.ac.uk/index.cfm; QCA: http://www.qca.org.uk). Data were obtained directly from teachers. At age 7 data are available for English and Mathematics; at ages 12 and 14 data are available for English, Mathematics, and Science. The teacher rating of English used a combined rating of students’ reading, writing, and speaking and listening; Mathematics used a combined score of knowledge in numbers, shapes, space, using and applying mathematics, and measures; and Science used a score combining life process, scientific enquiry, and physical process. These teacher ratings were found to be highly reliable when compared to the achievement measures collected by the UK National Pupil Database (NPD), as described later.

GCSE exam results were obtained from twins themselves or from their parents via questionnaires sent over mail or via telephone. GCSEs are UK-wide standardized examinations taken at age of 16 at the end of compulsory education. Children choose from a variety of different subjects, while English, Mathematics, and Science are compulsory. We used exam grades from English, Mathematics, and Science for the current analyses. Composite measures were created for English (mean of English language and English literature grades), Science (mean of single or double-weighted Science or, when taken separately Chemistry, Physics, and Biology grade), and Mathematics.

#### Measures of educational achievement obtained from the NPD

The TEDS dataset was linked to the NPD for every participant for whom we received written informed consent from either the twin or the parent. NPD is a rich UK database collecting data about students’ academic achievement across the school years (https://www.gov.uk/government/collections/national-pupil-database). Data are available for each Key Stage (KS) completed in the UK following the National Curriculum (NC). Teachers provide NC ratings for every student at the end of each KS (similarly to data collected at TEDS for the NC ratings in English, Mathematics, and Science). Exam scores as well as teacher ratings are available from KS1–KS3; and exam scores only are available for KS4 and KS5. Children’s ages for KS1, KS2, and KS3 are about 7, 11, and 14 years. KS4 marks the end of compulsory education with GCSE testing at about age 16. Sample size and descriptive characteristics for each measure are provided in Supplementary Table S1.

#### Composite scores of educational achievement

Composite scores were calculated at each KS combining the teacher ratings (both TEDS and NPD) with the exam scores for English, Mathematics, and Science separately by taking a mean of the three scores. The average correlation between NPD and TEDS teacher ratings was 0.70 (see Supplementary Table S5), and the average correlation between teacher ratings and exam scores was 0.80 (see Supplementary Table S6). For GCSE performance at the end of compulsory education, GCSE grades collected by TEDS and by NPD correlated 0.98 for English, 0.99 for Mathematics, and >0.95 for all Sciences. A mean score for NPD and TEDS was created to increase the sample size; when fewer measures were available we used any available data to calculate the composite score of educational achievement.

The overall achievement measure (core achievement) was calculated at each KS by taking a mean of English NC teacher ratings, Mathematics NC teacher rating (for both NPD and TEDS), English exam score, and Mathematics exam score. We did not include Science grades in overall achievement scores to make a more direct comparison across age because Science is not part of the National Curriculum at KS1.

#### Measures of general cognitive ability (*g*)

General cognitive ability (*g*; intelligence) was assessed in TEDS at ages 7, 9, 10, 12, 14, and 16. For the present analyses we created a longitudinal composite measure of *g* as a mean of these six assessments. See Supplementary Methods for a more detailed description of *g* measures.

### Analyses

#### Phenotypic analyses

The measures were described in terms of means and variance, comparing males and females and identical and non-identical twins; mean differences for age and sex and their interaction were tested using univariate ANOVA. Phenotypic correlations were calculated between academic achievement measures across development. The academic achievement measures were corrected for the small mean effects of age and sex (Supplementary Table S1) by rescoring the variable as a standardized residual correcting for age and sex, because in the analysis of twin data members of a twin pair are identical in age and MZ twins are identical for sex, and this would otherwise inflate twin estimates of shared environment.^55^ Full sex limitation genetic modeling has previously been reported for academic achievement and found only very minor sex differences in genetic and environmental estimates.^6,9,12^ For these reasons, and to increase power in the present analyses, the full sample was used, combining males and females and including opposite-sex pairs.

Finally, before conducting twin analyses, the achievement measures were corrected for skew because they were slightly negatively skewed. The achievement measures were corrected for skew by mapping it on to a standard normal distribution using the rank-based van der Waerden’s transformation.^56^

#### Twin design

The twin design was used for univariate and multivariate genetic analyses. The twin method offers a natural experiment capitalizing on the known genetic relatedness of MZ and DZ twin pairs. MZ twins are genetically identical and share 100% of their genes, while DZ twins share on average 50% of their segregating genes. Both MZ and DZ twins are assumed to share 100% of their shared environmental influences growing up in the same family. Non-shared environmental influences are unique to individuals, not contributing to similarity between twins. Using these known family relatedness coefficients, it is possible to estimate the relative contribution of additive genetic (A), shared environmental (C), and non-shared environmental (E) effects on the variance and covariance of the phenotypes, by comparing MZ correlations to DZ correlations. Heritability can be roughly calculated by doubling the difference between MZ and DZ correlations, C can be calculated by deducting heritability from MZ correlation and E can be estimated by deducting MZ correlation from unity (following Falconer’s formula).^47^ These parameters can be estimated more accurately using structural equation modeling, which also provides 95% confidence intervals and estimates of model fit. The structural equation modeling program OpenMx was used for all model-fitting analyses.^57^

These univariate analyses can be extended to multivariate analyses to study the etiology of covariance between multiple traits. Multivariate genetic method decomposes the covariance between traits into additive genetic (A), shared environmental (C), and non-shared environmental (E) components by comparing the cross-trait cross-twin correlations between MZ and DZ twin pairs. This method also enables estimation of the genetic correlation (*r*G), which is an index of pleiotropy, indicating the extent to which the same genetic variants influence two traits or measures of the same trait at two times. The shared environmental correlation (*r*C) and non-shared environmental correlation (*r*E) are estimated in a similar manner.^43,47^

We used two longitudinal models to study the issue of age-to-age stability of educational achievement.

The simplex model is a multivariate genetic model that estimates the extent to which the genetic and environmental influences on a trait are transmitted from age to age, and the extent to which innovative and age-specific influences emerge.^58^ The covariance or correlation matrix for such data is called simplex because the strength of the associations tends to correspond to differences between ages, that is, they are often highest along the diagonal and fall systematically as the difference between ages increases.^58^ The simplex model is illustrated in Supplementary Figure S2.

The common pathway model is a multivariate genetic model in which the variance common to all measures included in the analysis can be reduced to a common latent factor, for which the A, C, and E components are estimated. As well as estimating the etiology of the common latent factor, the model allows for the estimation of the A, C, and E components of the residual variance in each measure that is not captured by the latent construct.^59^ The common pathway model estimates the extent to which the stable variance in educational achievement across compulsory education (the latent factor of achievement) is explained by A, C, and E. The common pathway model is illustrated in Supplementary Figure S6.

#### SNP heritability

The genome-wide complex trait analysis (GCTA) software package enables estimates of the proportion of phenotypic variance or covariance that is explained by all SNPs that are available on genotype arrays, without testing the association of any single SNP individually.^17,49,60^ This estimate is often called SNP heritability. This method does not use known genetic relatedness coefficients but estimates heritability from DNA using only unrelated individuals. SNP heritability is calculated using restricted maximum likelihood and the variance and covariance is decomposed using mixed linear models.

First, the genetic relatedness matrix is calculated by weighting genetic similarities between all possible pairs of individuals with the allele frequencies across all SNPs on the DNA array. Individuals who are found to be even remotely related (greater than fifth cousins) are removed from the analyses as they would otherwise bias the results, which rely on chance genetic similarity between pairs of individuals.^17,18,61^ The matrix of pair-by-pair genetic similarity is compared to the matrix of pair-by-pair phenotypic similarity using the residual maximum likelihood estimation. SNP heritabilities were calculated for overall achievement across compulsory education, as well as for specific subjects.

#### Genome-wide polygenic scores

GPSs aggregate the effects of individual SNPs shown to be associated with the trait in a GWA study.^62^ GPSs were calculated for 6710 participants using summary statistics from Okbay et al.^28^ GWA analysis of years of education (*EduYears*).^28^ Of the 293,723 participants in the *EduYears* GWA discovery sample, the summary statistics excluded 23andMe participants, for legal reasons. Polygenic scores were constructed as the weighted sums of each individual’s genotype across all SNPs using the *LDpred* method^63^ (see Supplementary Methods for details). Delta *R*^2^ is reported as the estimate of variance explained by the GPS. These delta *R*^2^ estimates were obtained by comparing the incremental increase in the model *R*^2^ after adding the GPS to the regression model, and comparing this to the model that included 10 principal components in order to control for population stratification. See Supplementary Methods for genetic quality control and further information about GPS calculation.

We correlated *EduYears* with general educational achievement composites, as well as with performance in specific subjects at each age to estimate *EduYears* GPS heritability. Delta *R*^2^ are reported as the estimates of variance explained by adding the GPS to the regression model that included the academic achievement from all earlier ages to assess the extent to which *EduYears* contributes to age-to-age stability.

## Electronic supplementary material

Supplemental Material

Supplementary Methods

## Data Availability

For information on data availability, please see the TEDS data access policy. This can be found at: http://www.teds.ac.uk/research/collaborators-and-data/teds-data-access-policy. All relevant data are available from the authors according to the TEDS data access policy.

## Electronic supplementary material

**Supplementary information** accompanies the paper on the *npj Science of Learning* website (10.1038/s41539-018-0030-0).
