# Supplementary material for: The stability of educational achievement across school years is largely explained by genetic factors
Source: NPJ Sci Learn. 2018 Sep 4;3:16. doi: 10.1038/s41539-018-0030-0 (PMC6220264; doi:10.1038/s41539-018-0030-0)
Supplement: Supplementary file 1 — Supplemental Material [file 41539_2018_30_MOESM1_ESM.pdf]

Supplementary material

The stability of educational achievement across school years is largely explained by genetic factors

Kaili Rimfeld, Margherita Malanchini, Eva Krapohl, Laurie J. Hannigan, Philip S. Dale & Robert Plomin

Tables

Supplementary Table 1. Descriptive statistics. Mean (standard deviations in parentheses) for educational achievement

Supplementary Table 2. Twin intraclass correlations and model fitting results for univariate analyses for additive genetic (A), shared environmental (C), and non-shared environmental (E) components of variance for educational achievement for overall achievement, and for English, mathematics and science separately for teacher ratings and for exam grades (95% confidence intervals in parentheses)

Supplementary Table 3. Phenotypic and genetic correlations between school achievement across four ages for overall achievement (a), English (b), mathematics (c) and science (d). Phenotypic correlations are presented in the lower diagonal and genetic correlations in the upper diagonal (95% confidence intervals in parentheses)

Supplementary Table 4. (a)Variance explained by GPS (*EduYears*) across multiple achievement measures including teacher rating and exam performance and composite achievement measures for the following prior probabilities of fraction of casual markers 1.0; 0.1; 0.01; b) Variance explained by GPS (*EduYears*) across composite achievement measures and achievement in English, mathematics and science when controlling for *g* for the following prior probabilities of fraction of casual markers 1.0; 0.1; 0.01; (c) Variance explained by GPS (*EduYears*) across composite achievement measures and achievement in English, mathematics and Science when controlling for previous achievement for the following prior probabilities of fraction of casual markers 1.0; 0.1; 0.01; (d) c) Variance explained by GPS (*EduYears*) across composite achievement measures and achievement in English, mathematics and science when controlling for previous achievement and *g* for the following prior probabilities of fraction of casual markers 1.0; 0.1; 0.01

Supplementary Table 5. Correlations between teacher ratings collected at TEDS and national curriculum teacher assessment obtained from NPD (National Pupil Database) for English (a), mathematics (b) and science (c)

Supplementary Table 6. Phenotypic correlations between teacher ratings and TEDS and exam performance obtained from NPD (National Pupil Database) for English (a), mathematics (b) and science (c)

Figures

Supplementary Figure 1**.** (a) Twin model-fitting results for univariate analyses of educational achievement after correcting for intelligence using the regression method, (b) SNP heritability estimates of the proportion of variance explained by the additive effects of common SNPs (standard errors as error bars) for the same measures of educational achievement after correcting for intelligence.

Supplementary Figure 2. Simplex model

Supplementary Figure 3. Simplex model presenting the stability and change for English (a), mathematics (b) and science (c)

Supplementary Figure 4. Simplex model presenting the stability and change for the overall achievement (a), English (b), mathematics (c) and science (d) when only using standardized exam results

Supplementary Figure 5. Common pathway model

Supplementary Figure 6. (a) Variance explained by GPS (*EduYears*) in educational achievement across compulsory education (using the probabilities of fraction of casual markers 1); (b) Variance explained by GPS (*EduYears*) in educational achievement when controlling for *g* across compulsory education (using the probabilities of fraction of casual markers 1)

Supplementary Table 1. Descriptive statistics. Mean (standard deviations in parentheses) for educational achievement

Teacher ratings[a]		N	Whole sample	MZm[b]	MZf[b]	DZm[b]	DZf[b]	Dzos[b]	Sex[c]	Zygosity[c]	sex* zygosity[c]	R^2
**English**												
TEDS												
	KS1[e]	5745	.01 (0.99)	-0.21 (1.04)	0.08 (0.94)	-0.10 (1.07)	0.15 (0.93)	0.05 (0.97)	97.50**	13.90**	0.75	0.02
	KS2[e]	2819	0.02 (0.99)	-0.20 (1.02)	0.07 (0.96)	-0.06 (1.03)	0.11 (0.97)	0.08 (0.95)	53.32**	9.19**	0.06	0.02
	KS3[e]	498	0.15 (0.96)	-0.09 (0.95)	0.17 (1.04)	-0.18 (0.99)	0.01 (0.92)	0.05 (0.92)	5.77 *	0.04	0.33	0.01
NPD[d]												
	KS1	5280	2.23 (0.58)	2.13 (0.60)	2.24 (0.57)	2.21 (0.60)	2.27 (0.56)	2.25 (0.58)	39.44**	11.74**	0.21	0.01
	KS2	4798	4.30 (0.72)	4.14 (0.76)	4.30 (0.71)	4.24 (0.74)	4.39 (0.66)	4.34 (0.70)	49.00**	24.96**	0.2	0.01
	KS3	5067	5.75 (0.93)	5.57 (0.96)	5.85 (0.90)	5.60 (0.95)	5.88 (0.89)	5.77 (0.93)	103.96**	3.07	0.07	0.02
**Mathematics**												
TEDS												
	KS1	2818	0.01 (0.99)	-0.07 (1.08)	-0.06 (0.91)	0.05 (1.06)	-0.01 (0.95)	0.05 (0.99)	1.24	13.59**	2.31	<0.01
	KS2	2818	0.02 (0.99)	0.03 (1.04)	-0.08 (0.96)	0.09 (1.05)	-0.03 (0.96)	0.08 (0.95)	4.93*	3.83*	0.43	<0.01
	KS3	488	0.00 (1.03)	-0.14 (1.09)	0.03 (0.89)	0.08 (1.19)	-0.05 (1.08)	0.02 (0.97)	0.71	0.47	0.08	<0.01
NPD												
	KS1	5474	2.32 (0.57)	2.36 (0.57)	2.24 (0.55)	2.39 (0.58)	2.80 (0.55)	2.34 (0.57)	44.54**	5.68*	0.73	0.01
	KS2	4806	4.33 (0.74)	4.34 (0.78)	4.25 (0.74)	4.42 (0.75)	4.28 (0.72)	4.37 (0.71)	24.18**	9.41**	0.54	<0.01
	KS3	5132	6.33 (1.20)	6.37 (1.23)	6.24 (1.15)	6.44 (1.20)	6.25 (1.23)	6.36 (1.19)	7.61**	1.72	1.15	<0.01
**Science**												
TEDS												
	KS1	NA	NA									
	KS2	5786	0.02 (0.99)	-0.02 (1.07)	-0.06 (0.95)	0.05 (1.08)	0.03 (0.97)	0.07 (0.93)	0.01	5.47	1.12	<0.01
	KS3	480	0.02 (0.95)	0.03 (0.88)	0.07 (0.96)	0.03 (1.02)	-0.07 (0.96)	0.02 (0.97)	0.3	0.37	0.93	<0.01

NPD												
	KS1	5471	2.32 (0.55)	2.34 (0.57)	2.25 (0.52)	2.41 (0.56)	2.26 (0.51)	2.34 (0.55)	47.14**	6.72*	0.19	0.01
	KS2	4800	4.46 (0.65)	4.45 (0.69)	4.38 (0.68)	4.50 (0.65)	4.45 (0.63)	4.49 (0.62)	13.16**	13.91**	0.63	<0.01
	KS3	5132	6.33 (1.20)	5.94 (0.99)	5.87 (0.94)	5.99 (0.96)	5.91 (0.95)	5.96 (0.96)	10.97**	3.82	0.49	<0.01
**<u>Test scores</u>**												
**English**												
	KS1	5274	-0.07 (1.00)	-0.25 (1.10)	-0.03 (0.94)	-0.15 (1.04)	0.04 (0.91)	-0.03 (0.99)	58.06**	11.43**	0.01	0.01
	KS2	5407	64.88 (14.92)	62.22 (15.32)	65.62 (13.91)	63.31 (14.96)	66.92 (15.20)	65.32 (14.95)	67.44**	9.41**	0.02	0.01
	KS3	1199	52.61 (16.31)	50.32 (16.08)	53.22 (15.04)	49.58 (14.40)	54.53 (17.34)	53.61 (16.02)	14.31**	1.45	0.28	0.01
	GCSE	6317	8.91 (1.22)	8.65 (1.26)	9.06 (1.14)	8.75 (1.22)	9.09 (1.18)	8.92 (1.23)	159.16**	4.84*	0.16	0.03
**Mathematics**												
	KS1	5315	2.62 (0.47)	2.62 (0.49)	2.59 (0.48)	2.64 (0.51)	2.60 (0.44)	2.64 (0.46)	2.92	4.58*	0.76	<0.01
	KS2	5438	71.89 (19.56)	73.45 (19.81)	69.49 (19.23)	74.85 (18.99)	70.20 (19.96)	72.26 (19.43)	52.24**	2.77	0.00	0.01
	KS3	1220	85.20 (22.02)	88.37 (21.82)	83.85 (21.32)	85.89 (23.37)	81.50 (21.24)	86.75 (22.25)	12.90**	0.46	0.02	0.01
	GCSE	6268	8.91 (1.43)	8.97 (21.82)	8.84 (1.43)	9.01 (1.41)	8.88 (1.44)	8.91 (1.44)	10.50**	0.62	0	<0.01
**Science**												
	KS2	5446	62.89 (10.54)	62.90 (10.77)	61.95 (10.68)	63.77 (10.17)	62.96 (10.88)	63.04 (10.61)	10.00**	6.85**	0	<0.01
	KS3	1213	101.02 (23.32)	102.49 (22.54)	100.19 (21.50)	98.81 (22.03)	101.58 (23.08)	101.61 (22.55)	2.22	0.04	0.05	<0.01
	GCSE	5882	9.02 (1.29)	9.03 (1.29)	8.99 (1.30)	9.05 (1.29)	9.03 (1.33)	9.02 (1.29)	0.43	0.2	0.34	<0.01

Note: Raw scores, except for the composite scores that are standardized (see Methods)
[a] TEDS teacher assessments are done according to National Curriculum level grading (see Methods)
[b] MZm=monozygotic male; DZm=dizygotic male; MZf=monozygotic female; DZf=dizygotic female; DZos=dizygotic opposite sex
[c] Group difference = F statistics; *$p < 0.05$ **$p < 0.01$
[d] NPD– data obtained from the National Pupil Database (see Methods)
[e] KS1 age around 7; KS2 age around 11; KS3 age around 14, GCSE age around 16

Supplementary Table 2. Twin intraclass correlations and model fitting results for univariate analyses for additive genetic (A), shared environmental (C), and non-shared environmental (E) components of variance for educational achievement: for English, mathematics and science separately for teacher ratings and for test scores (95% confidence intervals in parentheses), and for composite achievement combining teacher ratings and test scores.

Teacher ratings[a]		Twin correlations MZ[b]	DZ	A	C	E
**English**						
TEDS						
	KS1[c]	.83 (.82-.85) (N=1957)	.50 (.50-.53) (N=3526)	.66 (.61-.71)	.18 (.13-.22)	.16 (.15-.17)
	KS2[c]	.80 (.78-.82) (N=950)	.50 (.46-.54) (N=1665)	.58 (.50-.75)	.22 (.15-.29)	.20 (19-.23)
	KS3[c]	.84 (.77-.89) (N=107)	.56 (.45-.65) (N=188)	.56 (.26-.77)	.26 (.05-.43)	.18 (.14-.24)
NPD[d]						
	KS1	.81 (.79-.82) (N=1859)	.45 (.42-.47) (N=3087)	.72 (.66-.77)	.09 (.04-.15)	.19 (.18-.21)
	KS2	.81 (.79-.82) (N=1684)	.43 (.40-.46) (N=2775)	.75 (.69-.81)	.05 (.01-.11)	.19 (.01-0.21)
	KS3	.74 (.72-.76) (N=1780)	.47 (.44-.50) (N=2928)	.53 (.47-.60)	.20 (.14-.26)	.26 (.25-.28)
**Mathematics**						
TEDS						
	KS1	.79 (.77-.80) (N=1936)	.47 (.44-.49) (N=3503)	.65 (.59-.70)	.14 (.09-.19)	.21 (.19-.22)
	KS2	.76 (.73-.79) (N=947)	.44 (.40-.47) (N=1656)	.64 (56-.73)	.12 (.04-.19)	.23 (.21-.26)
	KS3	.78 (.69-.85) (N=99)	.70 (.61-.77) (N=174)	.20 (.01-.38)	.58 (.42-.72)	.22 (.17-.30)
NPD						
	KS1	.77 (.75-.79) (N=1920)	.46 (.43-.48) (N=3214)	.63 (.58-.69)	.14 (.08-.19)	.22 (.21-.24)

	KS2	.81 (.80-.83) (N=1687)	.45 (.42-.48) (N=2775)	.71 (.65-.77)	.09 (.04-.15)	.19 (.18-.21)
	KS3	.82 (.81-.84) (N=1781)	.46 (.44-.49) (N=2939)	.73 (.68-.78)	.10 (.04-.15)	.17 (.16-.18)
**Science**						
TEDS						
	KS1	NA				
	KS2	.77 (.74-.79) (N=937)	.50 (.46-.53) (N=1627)	.53 (.45-.60)	.24 (.17-.31)	.23 (.21-.25)
	KS3	.80 (.71-.86) (N=103)	.54 (.43-.64) (N=178)	.55 (.34-.78)	.24 (.03-.42)	.21 (.16-.28)
NPD						
	KS1	NA				
	KS2	.80 (.79-.82) (N=1686)	.54 (.51-.56) (N=2773)	.51 (.46-.56)	.29 (.24-.37)	.20 (.19-.22)
	KS3	.76 (.74-.78) (N=1788)	.51 (.48-.54) (N=2934)	.50 (.44-.55)	.26 (.21-.31)	.24 (.23-.26)

Test scores[e]

**English**						
	KS1	.85 (.84-.86) (N=1858)	.48 (.46-.50) (N=3082)	.75 (.69-.80)	.10 (.05-.15)	.15 (.14-.16)
	KS2	.82 (.81-.84) (N=1870)	.51 (.49-.54) (N=3144)	.64 (.59-.69)	.19 (.14-.24)	.17 (.16-.18)
	KS3	.86 (.83-.88) (N=426)	.54 (.48-.59) (N=657)	.68 (.58-.79)	.19 (.08-.29)	.13 (.11-.15)
	GCSE	.83 (.81-.84) (N=2250)	.54 (.51-.55) (N=3962)	.59 (.55-.64)	.24 (.19-.28)	.17 (.16-.18)

**Mathematics**						
	KS1	.76 (.74-.78) (N=1858)	.47 (.45-.50) (N=3081)	.57 (.51-.63)	.19 (.14-.24)	.24 (.22-.26)
	KS2	.84 (.83-.86) (N=1889)	.46 (.43-.49) (N=3160)	.77 (.72-.83)	.07 (.02-.13)	.15 (.14-.16)
	KS3	.70 (.65-.75) (N=437)	.45 (.39-.51) (N=671)	.53 (.40-.66)	.18 (.06-.29)	.29 (.25-.34)
	GCSE	.82 (.81-.84) (N=2217)	.52 (.50-.54) (N=3922)	.61 (.56-.65)	.23 (.17-.26)	.17 (.16-.19)
**Science**						
	KS1	NA				
	KS2	.78 (.76-.80) (N=1893)	.50 (.47-.52) (N=3178)	.56 (.51-.62)	.21 (.16-.27)	.22 (.20-.23)
	KS3	.54 (.47-.60) (N=437)	.31 (.24-.38) (N=665)	.48 (.31-.60)	.06 (.01-.20)	.45 (.39-.52)
	GCSE	.83 (.81-.84) (N=2056)	.54 (.52-.56) (N=3535)	.57 (.53-.62)	.25 (.21-.30)	.17 (.16-.18)
**Composite achievement measures**						
**English Composite**[f]						
	KS1[a]	.87 (.86-.88) (N=2616)	.50 (.48-.52) (N=4651)	.74 (.70-.78)	.13 (.09-.17)	.13 (.12-.14)
	KS2	.85 (.84-.86) (N=2226)	.52 (.50-.54) (N=3824)	.66 (.62-.71)	.19 (.15-.23)	.15 (.14-.16)
	KS3	.77 (.75-.78) (N=1851)	.48 (.46-.51) (N=3062)	.56 (.50-.62)	.21 (.15-.26)	.23 (.22-.25)
**Mathematics Composite**[g]						

KS1	.81 (.79-.82)<br>(N=2623)	.46 (.44-.49)<br>(N=4674)	.70 (.65-.75)	.11 (.07-.15)	.19 (.18-.20)
KS2	.84 (.83-.86)<br>(N=2221)	.48 (.45-.50)<br>(N=3826)	.73 (.68-.78)	.11 (.07-.16)	.15 (.12-.16)
KS3	.82 (.80-.83)<br>(N=1845)	.47 (.45-.50)<br>(N=3058)	.70 (.65-.76)	.12 (.07-.17)	.17 (.16-.18)
**Science Composite**[h]					
KS1	NA				
KS2	.82 (.80-.83)<br>(N=2226)	.52 (.50-.54)<br>(N=3819)	.59 (.54-.63)	.23 (.19-.27)	.18 (.17-.19)
KS3	.74 (.72-.76)<br>(N=1848)	.49 (.47-.52)<br>(N=3052)	.50 (.45-.56)	.24 (.19-.29)	.25 (.24-.27)
**Core academic achievement**[i]					
KS1	.87 (.96-.88)<br>(N=2649)	.52 (.50-.54)<br>(N=4713)	.71 (.67-.75)	.16 (.12-.20)	.13 (.12-.13)
KS2	.88 (.87-.89)<br>(N=2236)	.53 (.51-.55)<br>(N=3845)	.70 (.66-.74)	.18 (.14-.22)	.12 (.11-.13)
KS3	.85 (.83-.86)<br>(N=1863)	.53 (.51-.56)<br>(N=3081)	.63 (.58-.68)	.22 (.17-.27)	.15 (.13-.16)
**Core academic achievement (test only)**[j]					
KS1	.85 (.84-.86)<br>(N=1859)	.51 (.48-.54)<br>(N=3083)	.69 (.64-.74)	.17 (.11-.21)	.15 (.14-.16)
KS2	.86 (.85-.87)<br>(N=1895)	.52 (.49-.54)<br>(N=3173)	.70 (.66-.75)	.17 (.12-.21)	.13 (.12-.14)

KS3	.83 (.80-.86) (N=443)	.54 (.48-.59) (N=676)	.62 (.52-.73)	.22 (.12-.31)	.16 (.14-.19)
GCSE	.87 (.86-.88) (N=2261)	.56 (.54-.58) (N=3996)	.61 (.57-.65)	.26 (.22-.30)	.13 (.12-.14)

Note: N= number of complete twin pairs
[a] TEDS teacher assessments are done according to National Curriculum level grading (see Methods)
[b] MZ=monozygotic; DZ=dizygotic
[c] KS1 age around 7; KS2 age around 11; KS3 age around 14, GCSE age around 16
[d] NPD– data obtained from the National Pupil Database (see Methods)
[e] Test performance obtained from National Pupil Database (see Methods)
[f] Mean score of English teacher ratings and test scores
[g] Mean score of mathematics teacher ratings and test score
[h] Mean score of Science teacher ratings and test scores
[i] Core academic achievement is computed as a mean of English and mathematics achievement
[j] Core academic achievement for tests only is computed as a mean of English test and mathematics test scores

Supplementary Table 3. Phenotypic and genetic correlations between school achievement across four ages for (a) Core achievement, (b) English, (c) mathematics and (d) science. Phenotypic correlations are presented in the lower diagonal and genetic correlations in the upper diagonal (95% confidence intervals in parentheses).

(a) Core achievement

	Achievement KS1	Achievement KS2	Achievement KS3	Achievement GCSE
Achievement KS1[b]	-	.87 (.86-.88)	.78 (.77-.79)	.72 (.71-.73)
Achievement KS2	.78 (.77 - .79)	-	.90 (.89 - .92)	.84 (.83 - .85)
Achievement KS3	.70 (.69 - .71)	.83 (.82 - .84)	-	.90 (.89 - .91)
Achievement GCSE	.66 (.64 - .67)	.79 (.78 - .80)	.85 (.84 - .86)	-

(b) English

	English KS1	English KS2	English KS3	English GCSE
English KS1	-	.83 (.82-.84)	.69 (.68-.70)	.66 (.65-.67)
English KS2	.75 (.74-.76)	-	.84 (.83-.85)	.80 (.79-.81)
English KS3	.62 (.61 - .63)	.74 (.73-.75)	-	.85 (.82-.86)
English GCSE	.60 (.59-.61)	.73 (.72-.73)	.75 (.74-.76)	-

(c) Mathematics

	Maths KS1	Maths KS2	Maths KS3	Maths GCSE
Mathematics KS1	-	.85 (.84-.85)	.78 (.76-.78)	.71 (.69-.72)
Mathematics KS2	.73 (.72-.74)	-	.91 (.89-.92)	.83 (.82-.84)
Mathematics KS3	.66 (.65 - .67)	.81 (.80 - .82)	-	.88 (.87-.89)
Mathematics GCSE	.61 (.60 - .62)	.75 (.74 - .76)	.80 (.79 - .81)	-

(d) Science

	Science KS2	Science KS3	Science GCSE
Science KS2	-	.84 (.83-.85)	.78 (.76-.82)
Science KS3	.68 (.67-.69)	-	.78 (.77-.79)
Science GCSE	.68 (.67-.69)	.71 (.70-.72)	-

[a] Core academic achievement is computed as a mean of English and mathematics achievement
[b] KS1 age around 7; KS2 age around 11; KS3 age around 14, GCSE age around 16

Supplementary Table 4. (a)Variance explained by GPS (*EduYears*) across multiple achievement measures including teacher rating and exam performance and composite achievement measures for the following prior probabilities of fraction of casual markers 1.0; 0.1; 0.01; b) Variance explained by GPS (*EduYears*) across composite achievement measures and achievement in English, mathematics and Science when controlling for *g* for the following prior probabilities of fraction of casual markers 1.0; 0.1; 0.01; (c) Variance explained by GPS (*EduYears*) across composite achievement measures and achievement in English, mathematics and science when controlling for previous achievement for the following prior probabilities of fraction of casual markers 1.0; 0.1; 0.01; (d) c) Variance explained by GPS (*EduYears*) across composite achievement measures and achievement in English, mathematics and science when controlling for previous achievement and *g* for the following prior probabilities of fraction of casual markers 1.0; 0.1; 0.01

(a)

**Teacher ratings**[a]		Causal fraction 0.01		Causal fraction 0.1		Causal fraction 1	
**English**		R^2	p	R^2	p	R^2	0
TEDS							
	KS1[b]	0.002355	1.41E-03	0.0333	2.20E-16	0.03403	2.20E-16
	KS2	0.008119	2.41E-05	0.03603	2.20E-16	0.0352	2.20E-16
	KS3	6.78E-03	0.08844	0.04013	2.92E-05	0.03871	4.07E-05
NPD[c]							
	KS1	0.001969	0.003919	0.02458	2.20E-16	0.02486	2.20E-16
	KS2	0.007494	8.00E-08	0.0393	2.20E-16	0.03795	2.20E-16
	KS3	0.00841	4.92E-09	0.06442	2.20E-16	0.06406	2.20E-16
**Mathematics**							
TEDS							
	KS1	0.002018	3.19E-03	0.02725	2.20E-16	0.02837	2.20E-16
	KS2	0.004356	0.002001	0.03318	2.20E-16	0.03382	2.20E-16
	KS3	0.004687	0.1707	0.0541	2.43E-06	0.05503	1.98E-06
NPD							
	KS1	0.001171	0.02356	0.02038	2.20E-16	0.02119	2.20E-16
	KS2	0.00558	3.66E-06	0.039	2.20E-16	0.03864	2.20E-16
	KS3	0.00760700	2.55E-08	0.06191	2.20E-16	0.06201	2.20E-16

**Science**							
TEDS							
	KS2	0.007712	4.16E-05	0.0285	2.36E-15	0.02957	7.07E-16
	KS3	0.008638	0.06039	0.05865	7.17E-07	0.05999	5.30E-07
NPD							
	KS2	0.0053	6.43E-06	0.03158	2.20E-16	0.03073	2.20E-16
	KS3	0.00754	2.95E-08	0.0648	2.20E-16	0.0656	2.20E-16
**Test scores[d]**		Causal fraction 0.01		Causal fraction 0.1		Causal fraction 1	
**English**							
	KS1	0.00458	1.08E-05	0.0319	2.20E-16	0.03117	2.20E-16
	KS2	0.007249	2.09E-08	0.04843	2.20E-16	0.04754	2.20E-16
	KS3	0.01027	0.001433	0.05574	5.73E-14	0.05269	2.88E-13
	GCSE	0.0145	2.20E-16	0.08874	8.87E-02	0.08849	2.20E-16
**Mathematics**							
	KS1	0.003206	0.0002332	0.03021	2.20E-16	0.03109	2.20E-16
	KS2	0.01013	3.10E-11	0.05288	5.29E-02	0.05244	2.20E-16
	KS3	0.009029	0.002554	0.03769	5.33E-10	0.03891	2.78E-10
	GCSE	0.01142	2.34E-13	0.08492	2.20E-16	0.08681	2.20E-16
**Science**							
	KS2	0.007058	2.94E-08	0.04517	2.20E-16	0.04465	2.20E-16
	KS3	0.007058	2.94E-08	0.01484	1.09E-04	0.01367	2.05E-04
	GCSE	0.011910	3.70E-13	0.08551	2.20E-16	0.08557	2.20E-16

**Composite achievement measures**						
**English Composite[e]**						
KS1	0.004172	1.27E-06	0.03444	2.20E-16	0.03472	2.20E-16
KS2	0.009493	6.51E-12	0.05048	2.20E-16	0.04953	2.20E-16
KS3	0.007657	1.23E-08	0.06576	2.20E-16	0.06535	2.20E-16
**Mathematics Composite[f]**						
KS1	0.003113	2.77E-05	0.03115	2.20E-16	0.03199	2.20E-16
KS2	0.007979	3.02E-10	0.05246	2.20E-16	0.05218	2.20E-16
KS3	0.007756	1.02E-08	0.0611	2.20E-16	0.06164	2.20E-16
**Science Composite[g]**						
KS2	0.008213	1.69E-10	0.04454	4.45E-02	0.04426	2.20E-16
KS3	0.007569	1.53E-08	0.05981	2.20E-16	0.05995	2.20E-16
**Core academic achievement[h]**						
KS1	0.00421	1.02E-06	0.03776	2.20E-16	0.03846	2.20E-16
KS2	0.01002	1.58E-12	0.05807	2.20E-16	0.05734	2.20E-16
KS3	0.01028	3.70E-11	0.07753	2.20E-16	0.07736	2.20E-16
**Core academic achievement (test only)[i]**						
KS1	0.00504	3.88E-06	0.03812	2.20E-16	0.03797	2.20E-16
KS2	0.01024	2.27E-11	0.06055	2.20E-16	0.05963	2.20E-16
KS3	0.01191	1.19E-02	0.06308	4.72E-16	0.06182	9.45E-16
GCSE	0.01502	2.20E-16	0.1036	2.20E-16	0.1044	2.20E-16
**At least 5 GCSEs grades A*-C**						
	0.004117	1.35E-05	0.03735	2.20E-16	0.0391	2.20E-16
**University Yes/No**						
	0.007047	5.31E-09	0.04382	2.20E-16	0.04482	2.20E-16

(b)

Core achievement after controlling for *g*						
KS1	0.00077108	0.0221874	0.007163709	2.81E-12	0.007267692	1.96E-12
KS2	0.003240369	2.46E-06	0.01284831	4.66E-21	0.01281044	5.32E-21
KS3	0.003432505	6.75E-06	0.02347825	1.70E-32	0.02329252	3.02E-32
GCSE	0.006762447	4.48E-11	0.03809764	1.96E-56	0.03881604	1.70E-57
**English Composite**						
KS1	0.000644961	0.04042662	0.005992973	3.89E-10	0.005962811	4.31E-10
KS2	0.003274555	4.88E-06	0.01052223	2.14E-16	0.01025046	5.21E-16
KS3	0.002810447	1.49E-04	0.02175303	2.34E-26	0.02121292	9.81E-26
GCSE	0.007940575	4.92E-13	0.03320625	2.54E-50	0.03312769	3.33E-50
**Mathematics Composite**						
KS1	0.000612717	0.04792755	0.006014177	5.36E-10	0.00619134	2.99E-10
KS2	0.002185918	1.79E-04	0.01215049	7.58E-19	0.01244495	2.86E-19
KS3	0.002046046	8.01E-04	0.01672076	5.57E-22	0.01718508	1.49E-22
GCSE	0.004561106	4.03E-08	0.03032397	2.56E-46	0.03173265	1.95E-48
**Science Composite**						
KS2	0.002632976	3.04E-05	0.007573711	1.38E-12	0.007791726	6.56E-13
KS3	0.002152566	0.000848456	0.01728384	1.99E-21	0.01755325	9.69E-22
GCSE	0.00611269	4.47E-10	0.03350121	2.95E-49	0.03324061	7.05E-49

(c)

Achievement controlling for previous achievement						
	Causal fraction 0.01		Causal fraction 0.1		Causal fraction 1	
**Core achievement**	R^2	p	R^2	p	R^2	p
KS2	0.002206686	1.93E-07	0.008475095	1.25E-24	0.008183667	7.88E-24

KS3	0.000383142	2.39E-02	0.00592282	4.59E-19	0.006034617	2.13E-19
GCSE	0.00149244	6.86E-07	0.004583105	2.58E-18	0.004608528	2.08E-18
**English Composite**						
KS2	0.002058892	2.58E-06	0.007181993	1.28E-18	0.006811978	9.85E-18
KS3	0.000471534	3.93E-02	0.008811997	3.45E-19	0.008846821	2.94E-19
GCSE	0.002277141	5.95E-07	0.005983939	4.89E-16	0.005875914	8.97E-16
**Mathematics Composite**						
KS2	0.002421696	9.26E-07	0.01044621	1.40E-24	0.01017681	5.55E-24
KS3	0.00023299	0.09764897	0.003644199	5.14E-11	0.003827579	1.69E-11
GCSE	0.000765744	0.001199653	0.004770229	5.00E-16	0.005005869	9.48E-17
**Science Composite**						
KS3	0.000730478	0.02071688	0.01090635	2.74E-19	0.0110114	1.85E-19
GCSE	0.002754109	1.20E-06	0.01270403	9.83E-26	0.0126376	1.32E-25

(d)

Achievement controlling for previous achievement and g

	Causal fraction 0.01		Causal fraction 0.1		Causal fraction 1	
**Core achievement**	R^2	p	R^2	p	R^2	p
KS2	0.001536341	1.75E-05	0.004090731	2.19E-12	0.003982051	4.29E-12
KS3	0.000497907	1.53E-02	0.005366822	1.31E-15	0.005246497	2.73E-15
GCSE	0.00109586	7.12E-05	0.004087361	1.42E-14	0.004194769	6.39E-15
**English Composite**						
KS2	0.001645886	4.00E-05	0.003150068	1.29E-08	0.00293433	4.04E-08
KS3	0.000687653	1.93E-02	0.007746146	3.13E-15	0.007429777	1.15E-14
GCSE	0.002080597	6.81E-06	0.005070878	1.94E-12	0.005038167	2.28E-12
**Mathematics Composite**						
KS2	0.001114738	9.23E-04	0.004529811	2.21E-11	0.004567237	1.82E-11

KS3	0.000190717	0.15654	0.002621455	1.42E-07	0.002721337	8.23E-08
GCSE	0.000389761	0.03176443	0.004103637	2.76E-12	0.00438947	4.81E-13
**Science Composite**						
KS3	0.000562775	0.05436984	0.007736536	8.26E-13	0.007724215	8.61E-13
GCSE	0.002054132	6.07E-05	0.01048105	8.75E-20	0.01031169	1.74E-19

[a] TEDS teacher assessments are done according to National Curriculum level grading (see Methods)
[b] KS1 age around 7; KS2 age around 11; KS3 age around 14, GCSE age around 16
[c] NPD– data obtained from the National Pupil Database (see Methods)
[d] Test performance obtained from National Pupil Database (see Methods)
[e] Mean score of English teacher ratings and test scores
[f] Mean score of mathematics teacher ratings and test score
[g] Mean score of Science teacher ratings and test scores
[h] Core academic achievement is computed as a mean of English and mathematics achievement
[i] Core academic achievement for tests only is computed as a mean of English test and mathematics tests

Supplementary Table 5. Correlations between teacher ratings collected at TEDS and national curriculum teacher assessment obtained from NPD (National Pupil Database) for English (a), mathematics (b) and science (c).

a) English

	TEDS KS1 English	NPD KS1 English	TEDS KS2 English	NPD KS2 English	TEDS KS3 English	NPD KS3 English
TEDS KS1 English	1					
NPD KS1 English	.770**	1				
TEDS KS2 English	.643**	.598**	1			
NPD KS2 English	.620**	.598**	.659**	1		
TEDS KS3 English	.571**	.502**	.624**	.617**	1	
NPD KS3 English	.560**	.523**	.590**	.629**	.725**	1

b) Mathematics

	TEDS KS1 mathematics	NPD KS1 mathematics	TEDS KS2 mathematics	NPD KS2 mathematics	TEDS KS3 mathematics	NPD KS3 mathematics
TEDS KS1 mathematics	1					
NPD KS1 mathematics	.710**	1				
TEDS KS2 mathematics	.594**	.551**	1			
NPD KS2 mathematics	.594**	.593**	.661**	1		
TEDS KS3 mathematics	.543**	.510**	.654**	.656**	1	
NPD KS3 mathematics	.587**	.597**	.654**	.743**	.796**	1

c) Science

	TEDS KS2 science	NPD KS2 science	TEDS KS3 science	NPD KS3 science
TEDS KS2 science	1			
NPD KS2 science	.538**	1		
TEDS KS3 science	.512**	.573**	1	
NPD KS3 science	.524**	.636**	.733**	1

Note: **p < 0.001
Note: KS1 age around 7; KS2 age around 11; KS3 age around 14, GCSE age around 16

Supplementary Table 6. Phenotypic correlations between teacher ratings and TEDS and exam performance obtained from NPD (National Pupil Database) for English (a), mathematics (b) and science (c).

a) **English**

	TEDS KS1 Teacher ratings English	NPD KS1 Test scores English	TEDS KS2 Teacher ratings English	NPD KS2 Test scores English	TEDS KS3 teacher ratings English	NPD KS3 Test scores English
TEDS KS1 Teacher ratings English	1					
NPD KS1 Test scores English	.801**	1				
TEDS KS2 Teacher ratings English	.643**	.692**	1			
NPD KS2 Test scores English	.643**	.721**	.676**	1		
TEDS KS3 teacher ratings English	.571**	.580**	.624**	.643**	1	
NPD KS3 Test scores English	.587**	.647**	.643**	.745**	.664**	1

b) **Mathematics**

	TEDS KS1 Teacher ratings maths	NPD KS1 Test scores maths	TEDS KS2 Teacher ratings maths	NPD KS2 Test scores maths	TEDS KS3 Teacher ratings maths	NPD KS3 Test scores maths
TEDS KS1 Teacher ratings maths	1					
NPD KS1 Test scores maths	.687**	1				
TEDS KS2 Teacher ratings maths	.594**	.620**	1			
NPD KS2 Test scores maths	.616**	.709**	.695**	1		
TEDS KS3 Teacher ratings maths	.543**	.543**	.654**	.747**	1	
NPD KS3 Test scores maths	.457**	.490**	.545**	.602**	.556**	1

c) Science

	TEDS KS2 Teacher ratings science	NPD KS2 Test scores science	TEDS KS3 Teacher ratings science	NPD KS3 Test scores science
TEDS KS2 Teacher ratings science	1			
NPD KS2 Test scores science	.534**	1		
TEDS KS3 Teacher ratings science	.512**	.643**	1	
NPD KS3 Test scores science	.241**	.251**	.194**	1

Note: **p < 0.001
Note: KS1 age around 7; KS2 age around 11; KS3 age around 14, GCSE age around 16

Supplementary Figure 1. (a) Twin model-fitting results for univariate analyses of educational achievement after correcting for intelligence using the regression method. A=additive genetic; C=shared environmental; E=non-shared environmental proportions of the variance. (b) SNP heritability estimates of the proportion of variance explained by the additive effects of common SNPs (standard errors as error bars) for the same measures of educational achievement after correcting for intelligence. SNP heritabilities were calculated following adjustment for sex and population stratification.

(a)

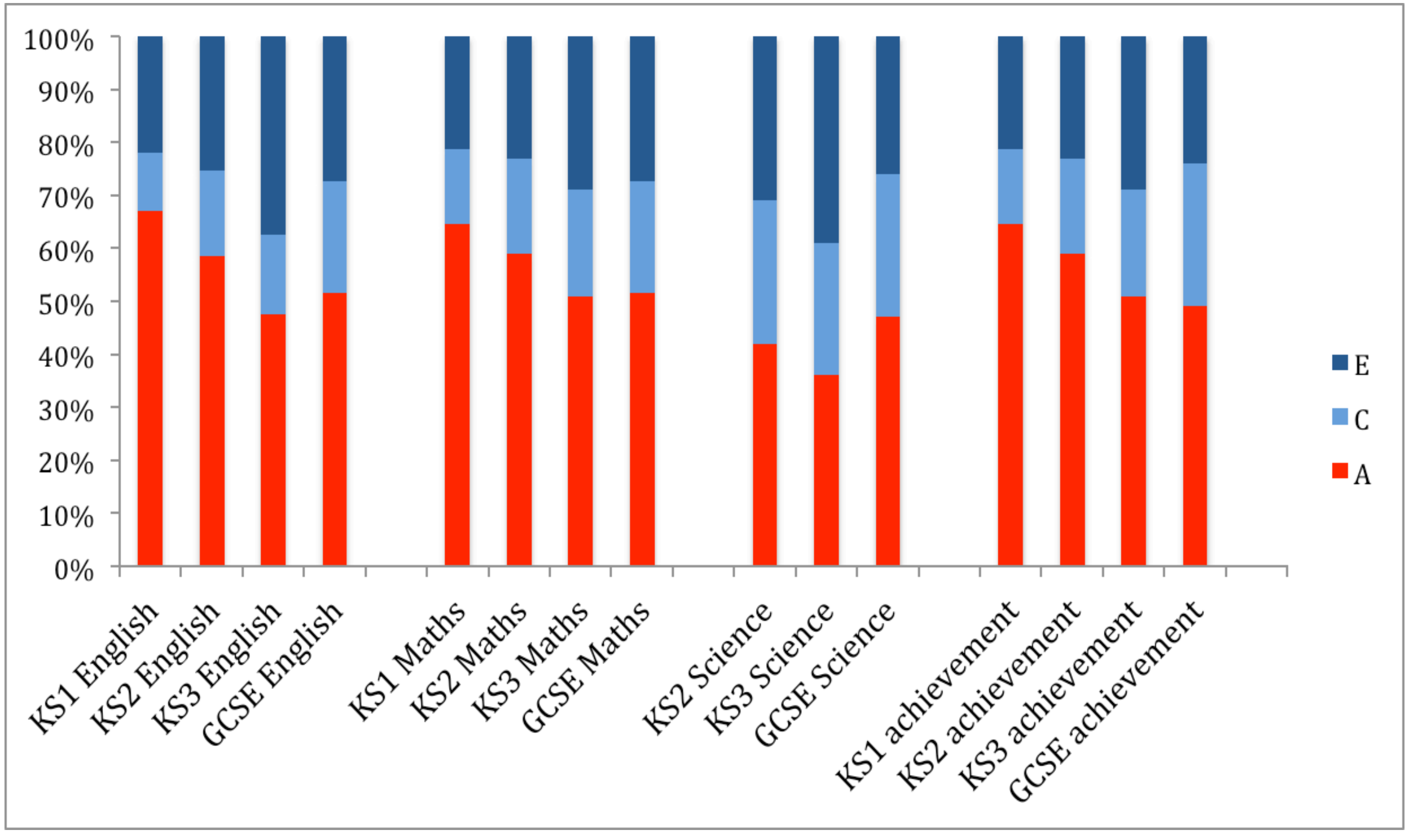

(b)

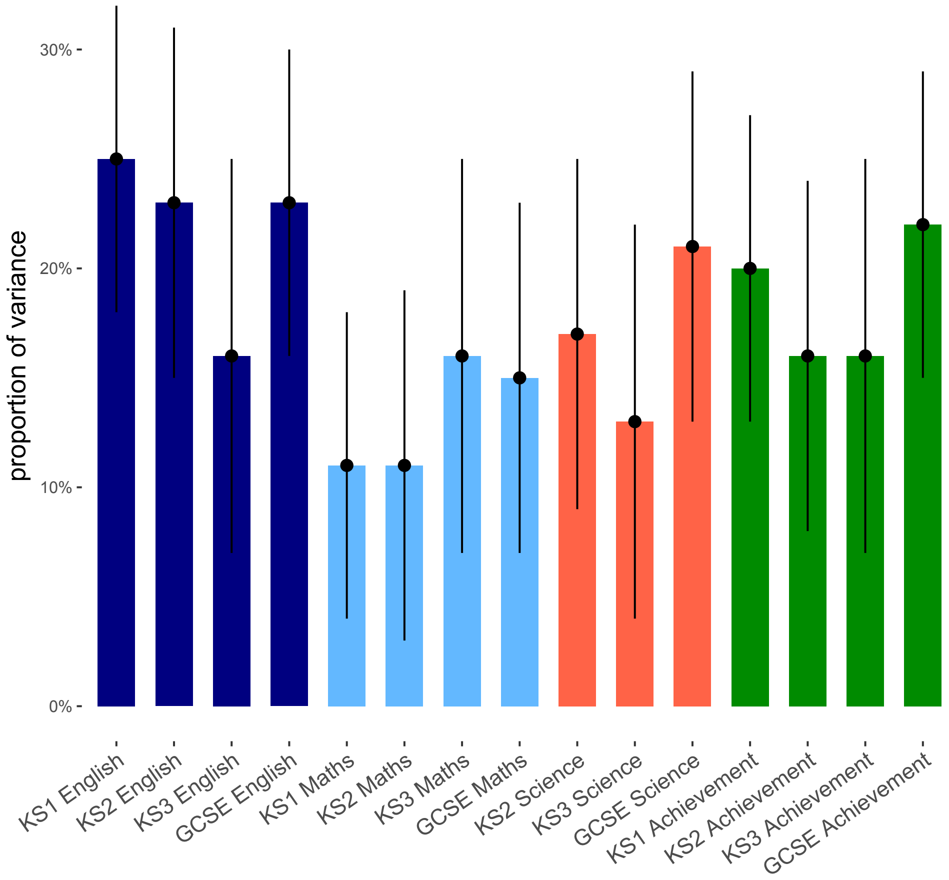

Note: KS1 age around 7; KS2 age around 11; KS3 age around 14, GCSE age around 16
Note: Core academic achievement is computed as a mean of English and mathematics achievement

Supplementary Figure 2. Genetic simplex model

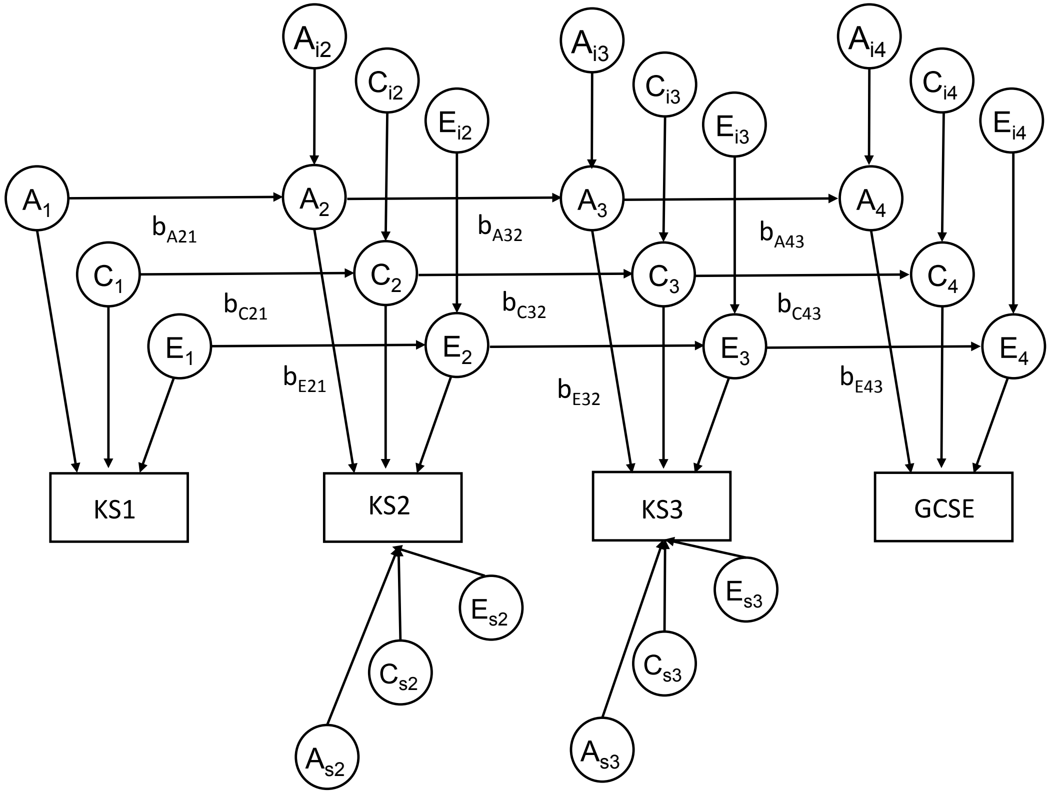

Genetic simplex model is a longitudinal model that estimates the additive genetic (A) , shared environmental (C) and non-shared environmental (E) proportions of variance over time; genetic (environmental) transmission is estimated and presented with beta values (for example b_{A21} presents the genetic transmission from KS1 to KS2); new genetic (environmental) variance is introduced at each time point (innovation; i) and these effects are then transmitted to next measurement occasion; additionally there are time specific genetic (environmental) influences that are nor transmitted to next measurement occasion (specific influences; s).

Supplementary Figure 3. Simplex model presenting the stability and change for (a), English (Eng), (b) mathematics (Mat) and (c) science (Sci)

(a)

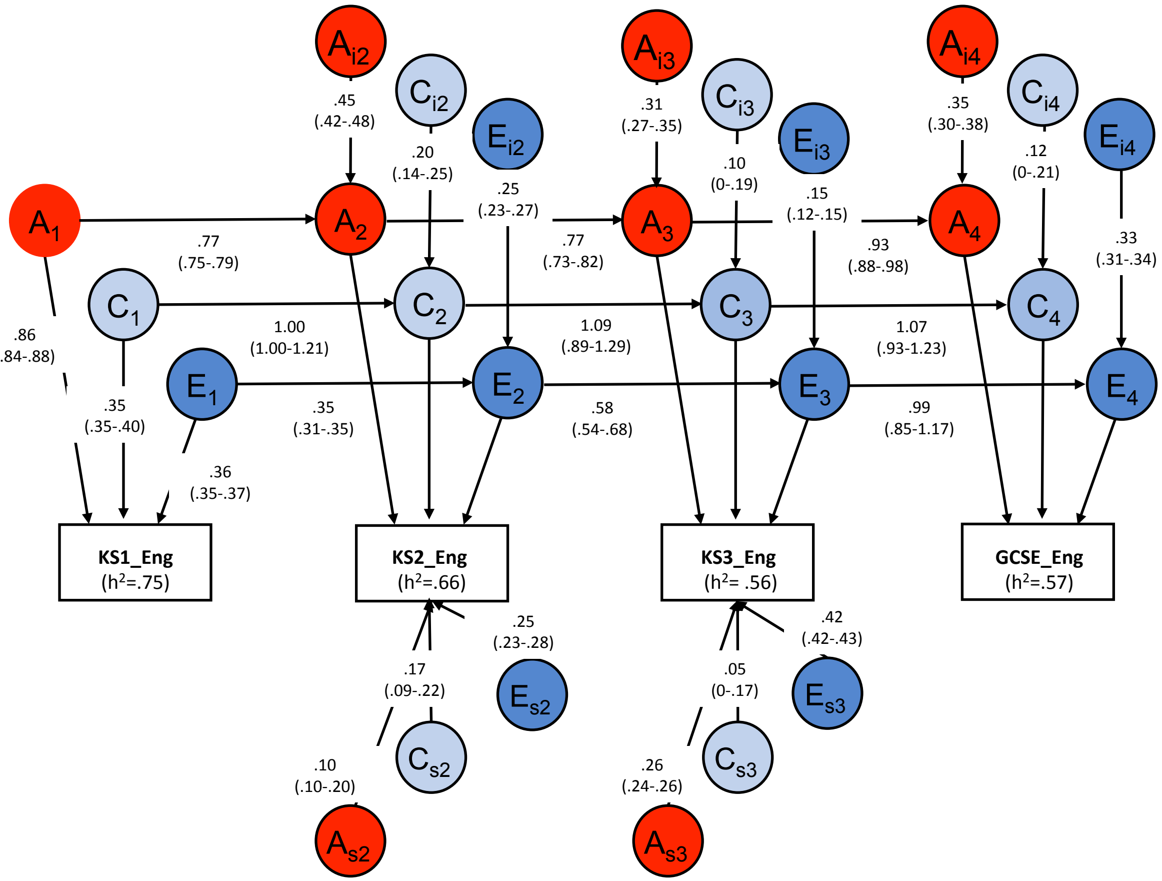

(b)

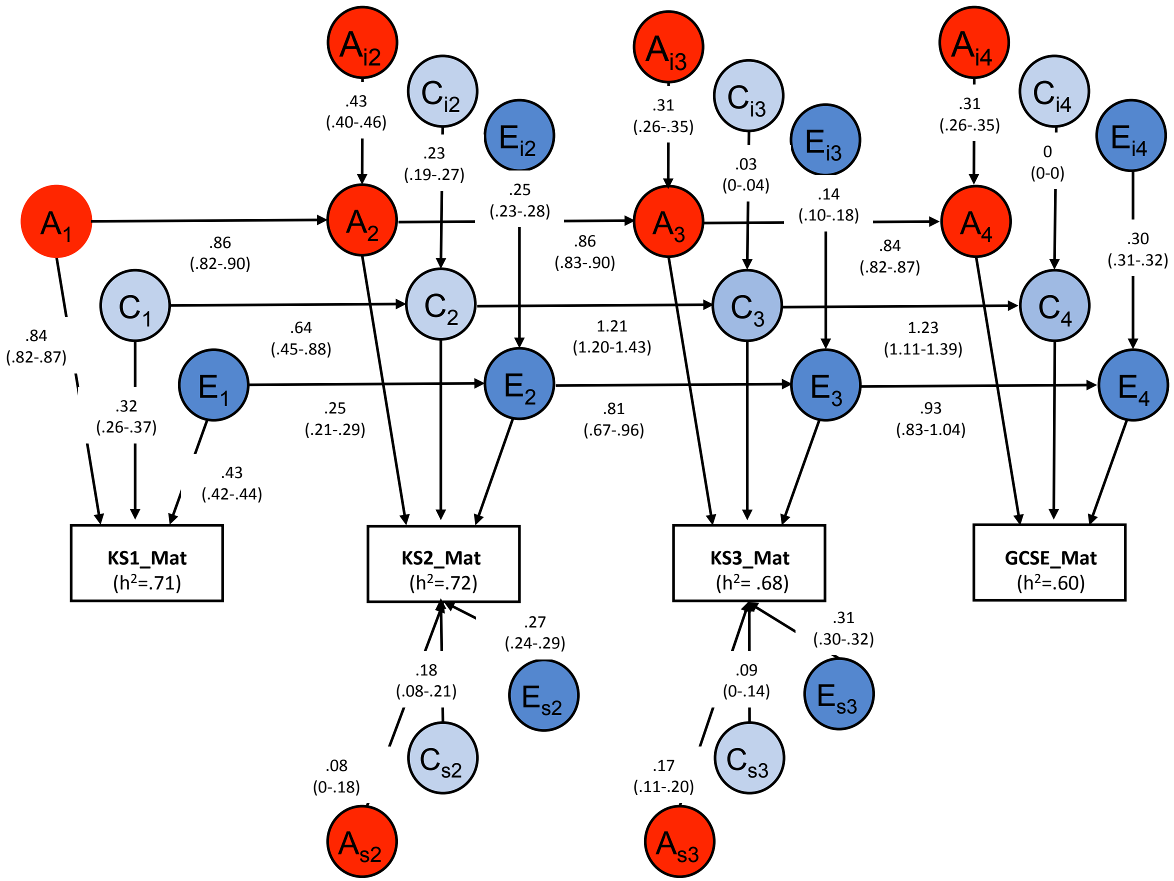
A1
A2
A3
A4
Ai2
Ai3
Ai4
Ci2
Ci3
Ci4
Ei2
Ei3
Ei4
C1
C2
C3
C4
E1
E2
E3
E4
As2
As3
Cs2
Cs3
Es2
Es3
.43 (.40-.46)
.23 (.19-.27)
.25 (.23-.28)
.31 (.26-.35)
.03 (0-.04)
.14 (.10-.18)
.31 (.26-.35)
0 (0-0)
.30 (.31-.32)
.86 (.82-.90)
.86 (.83-.90)
.84 (.82-.87)
.84 (.82-.87)
.64 (.45-.88)
1.21 (1.20-1.43)
1.23 (1.11-1.39)
.32 (.26-.37)
.25 (.21-.29)
.81 (.67-.96)
.93 (.83-1.04)
.43 (.42-.44)
KS1_Mat (h²=.71)
KS2_Mat (h²=.72)
KS3_Mat (h²= .68)
GCSE_Mat (h²=.60)
.27 (.24-.29)
.18 (.08-.21)
.08 (0-.18)
.31 (.30-.32)
.09 (0-.14)
.17 (.11-.20)

(c)

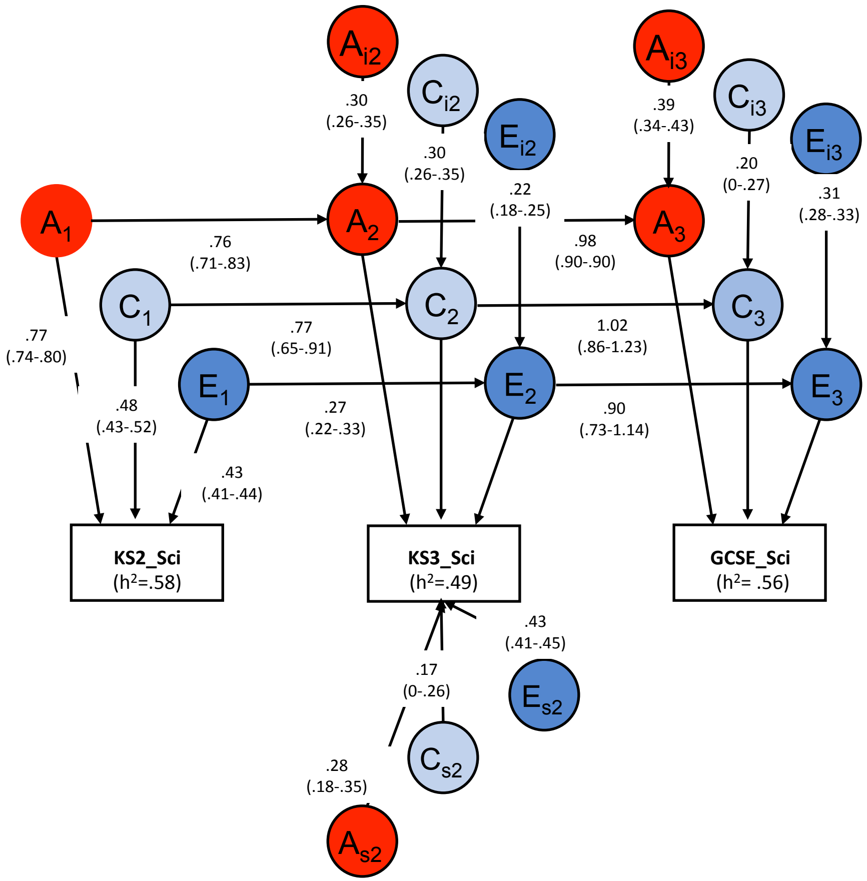

Note: KS1 age around 7; KS2 age around 11; KS3 age around 14, GCSE age around 16

Supplementary Figure 4. Simplex model presenting the stability and change for core achievement when only using standardized tests scores results

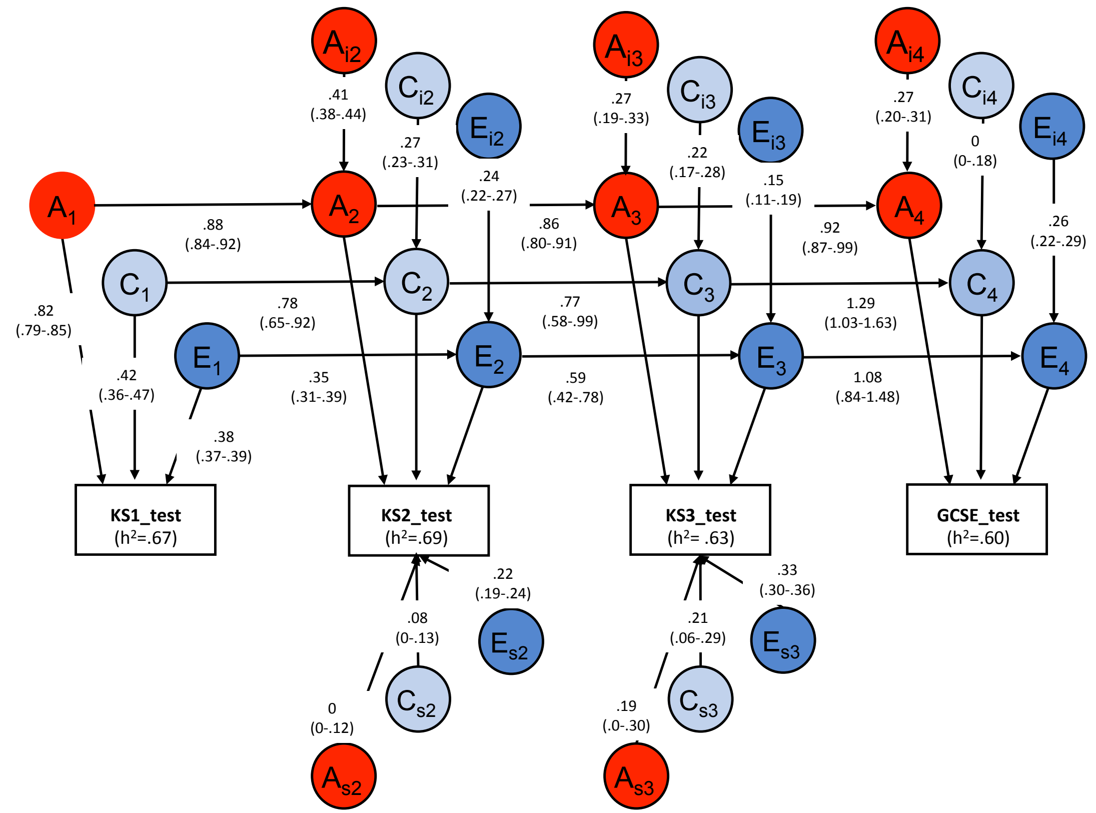

Note: KS1 age around 7; KS2 age around 11; KS3 age around 14, GCSE age around 16
Note: Core academic achievement is computed as a mean of English and mathematics achievement

Supplementary Figure 5. Common pathway model; a_l, c_l, e_l = path coefficients for A, C and E common influences on the common latent factor; f_1, f_2, f_3, f_4 = path coefficients for the influence of the common etiological factor on each construct; a_s, c_s, e_s, = path coefficients for specific sources of A, C and E variance on each construct.

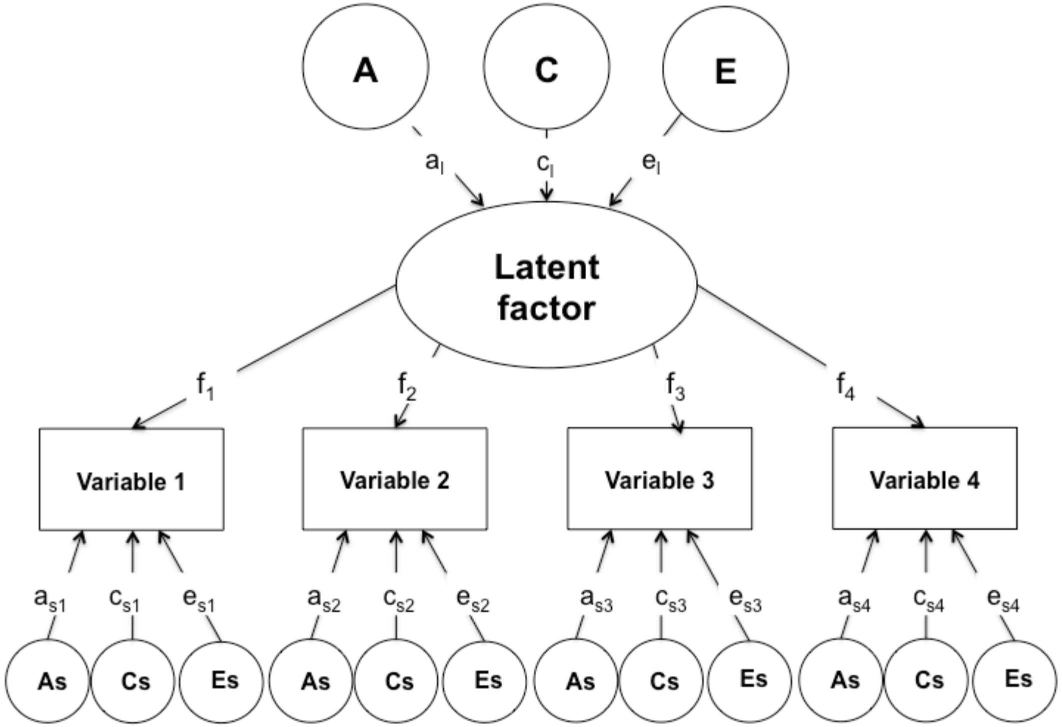

The common pathway model is a multivariate genetic model in which the etiology of all the variables in the analysis can be reduced to a common latent factor. That is, all genetic, shared environmental and non-shared environmental influences on all observed variables in the analyses will load onto a single latent factor. The model then estimates the ACE of common latent factor and residual paths index the extent to which the variance of the traits is not shared with the common factor (As, Cs, Ec); model also estimates the factor loadings to the common latent factor (f_1-f_4)

Supplementary Figure 6. (a) Variance explained by GPS (*EduYears*) in educational achievement across compulsory education (See Supplementary methods) (b) Variance explained by GPS (*EduYears*) in educational achievement when controlling for *g* across compulsory education (See Table S4 for full results)

(a)

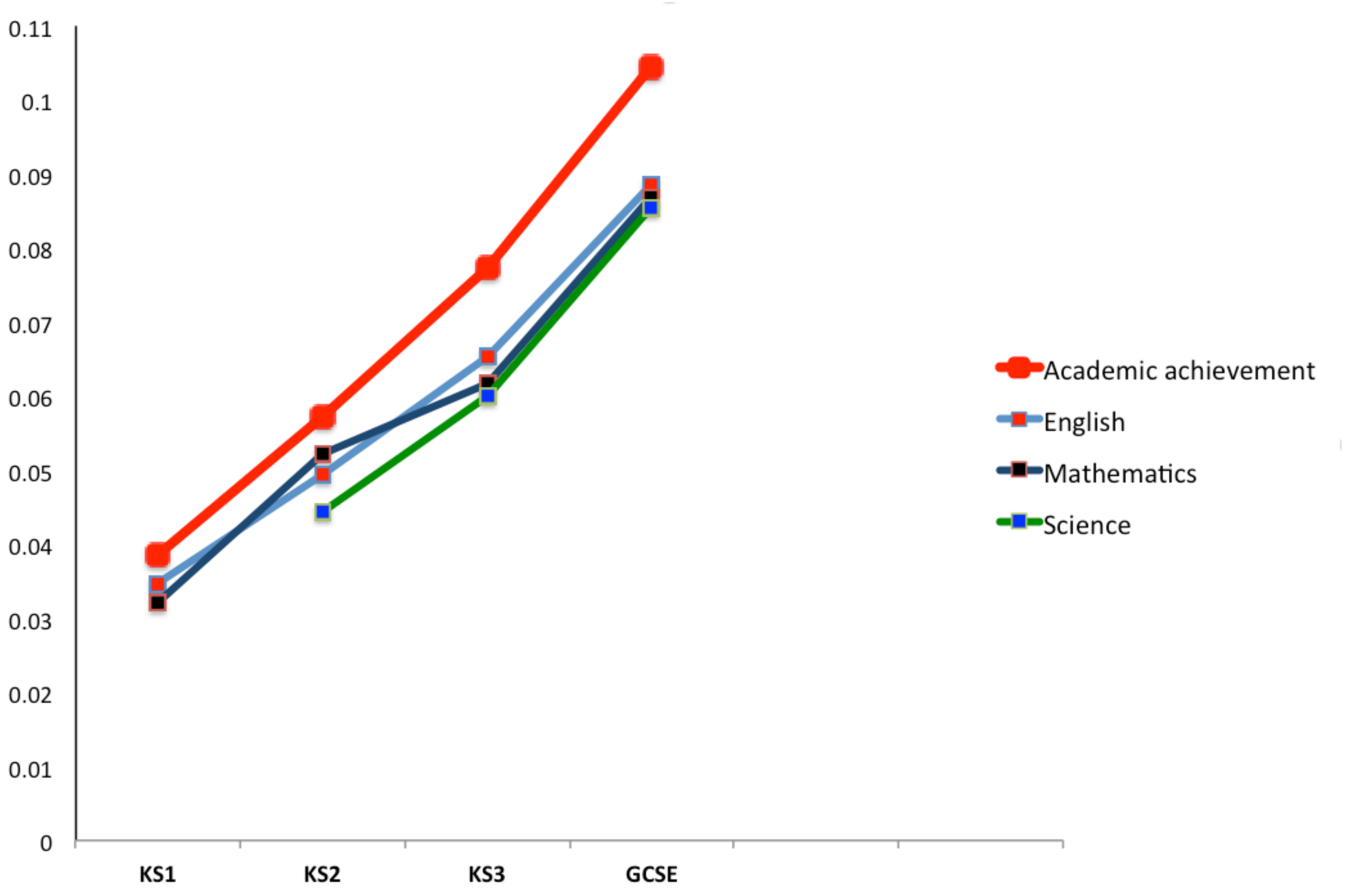

(b)

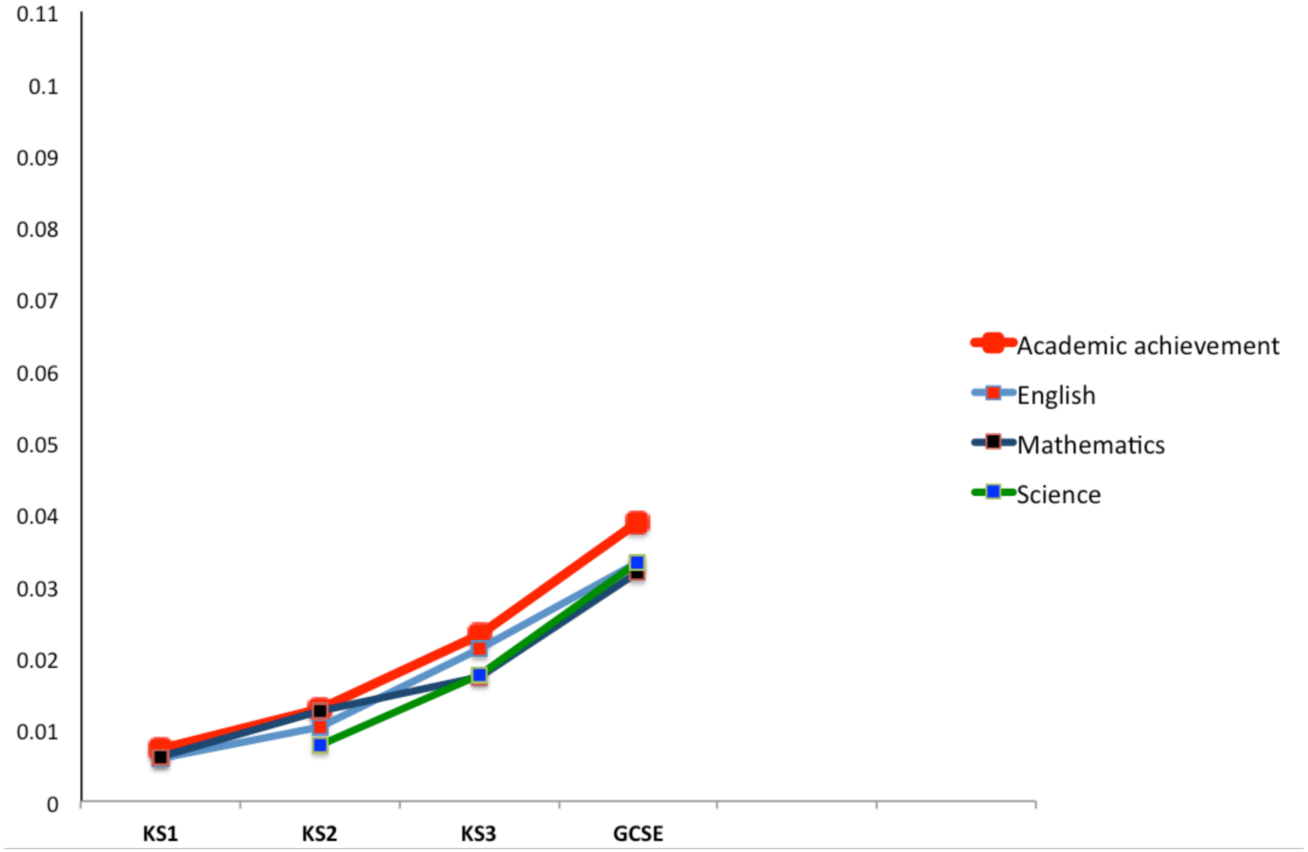

Note: KS1 age around 7; KS2 age around 11; KS3 age around 14, GCSE age around 16;
Core academic achievement is computed as a mean of English and mathematics achievement
