# Supplementary material for: The stability of educational achievement across school years is largely explained by genetic factors
Source: NPJ Sci Learn. 2018 Sep 4;3:16. doi: 10.1038/s41539-018-0030-0 (PMC6220264; doi:10.1038/s41539-018-0030-0)
Supplement: Supplementary file 2 — Supplementary Methods [file 41539_2018_30_MOESM2_ESM.pdf]

Supplementary Methods

The stability of educational achievement across school years is largely explained by genetic factors

Kaili Rimfeld, Margherita Malanchini, Eva Krapohl, Laurie J. Hannigan, Philip S. Dale & Robert Plomin

Genotyping protocol and quality control and imputation

DNA for 4,649 individuals was extracted from saliva and buccal cheek swab samples and hybridized to HumanOmniExpressExome-8v1.2 genotyping arrays at the Institute of Psychiatry, Psychology and Neuroscience Genomics & Biomarker Core Facility. The raw image data from the array were normalized, pre-processed, and filtered in GenomeStudio according to Illumina Exome Chip SOP v1.4. (http://confluence.brc.iop.kcl.ac.uk:8090/display/PUB/Production+Version%3A+Illumina+Exome+Chip+SOP+v1.4). In addition, prior to genotype calling, 869 multi-mapping SNPs and 353 samples with callrate <.95 were removed. The ZCALL program was used to augment the genotype calling for samples and SNPs that passed the initial QC.

DNA from 3,665 samples was extracted from buccal cheek swabs and genotyped at Affymetrix, Santa Clara, California, USA. Samples were successfully hybridized to AffymetrixGeneChip 6.0 SNP genotyping arrays (http://www.affymetrix.com/support/technical/datasheets/genomewide_snp6_datasheet.pdf) using experimental protocols recommended by the manufacturer (Affymetrix Inc., Santa Clara, CA). The raw image data from the arrays were normalized and pre-processed at the Wellcome Trust Sanger Institute, Hinxton, UK for genotyping as part of the Wellcome Trust Case Control Consortium 2 (https://www.wtccc.org.uk/ccc2/) according to the manufacturer's

guidelines (http://www.affymetrix.com/support/downloads/manuals/genomewidesnp6_manual.pdf). Genotypes for the Affymetrix arrays were called using CHIAMO (https://mathgen.stats.ox.ac.uk/genetics_software/chiamo/chiamo.html).

After initial quality control and genotype calling, the same quality control was performed on the samples genotyped on the Illumina and Affymetrix platforms separately using PLINK[1,2], R[3], and vcftools[4].

Samples were removed from subsequent analyses based on call rate (<0.99), suspected non-European ancestry, heterozygosity, array signal intensity, and relatedness. SNPs were excluded if the minor allele frequency was <0.5%, if more than 1% of genotype data were missing, or if the Hardy Weinberg p-value was lower than 10^{-5}. Non-autosomal markers and indels were removed. Association between the SNP and the platform, batch, or plate on which samples were genotyped was calculated; SNPs with an effect p-value less than 10^{-3} were excluded. A total sample of 6,710 samples, with 3,617 individuals and 600,034 SNPs genotyped on Illumina and 3,093 individuals and 525,859 SNPs genotyped on Affymetrix remained after quality control.

Genotypes from the two platforms were separately imputed using the Haplotype Reference Consortium[5] and Minimac3 1.0.13[6,7] available on the *Michigan Imputation Server* as reference data. A series of quality checks was performed before merging data from the two platforms' imputation (e.g. platform effects, allele frequencies by imputation quality). For the present analyses, we limited our analyses to variants genotyped or imputed at info >.70 on both platforms, allele frequency difference between platforms smaller than 5%, and Hardy Weinberg p-value was greater than 10^{-5}. Using these criteria, 7,581,516 genotyped and well-imputed SNPs were retained for the analyses.

We performed principal component analysis on a subset of 26,385 common (MAF>5%) autosomal HapMap3 SNPs [8], after stringent pruning to remove markers in linkage disequilibrium ($r^2 > 0.05$) and excluding high linkage disequilibrium genomic regions to ensure that only genome-wide effects were detected.

Polygenic score model

Using summary statistics from *EduYears* GWA study[9], we constructed polygenic scores as the weighted sums of the individual's genotype across all SNPs. The scores are calculated as the weighted sums of individual *i*'s SNPs: $GPS_{ki} = \sum_{j=i}^{m} \hat{\beta}_{kj} g_{kji}$

GPS_{ik} represents the individual i's polygenic score based on summary statistics from $GWAS_k$. $\hat{\beta}_{kj}$ is an estimate of marker j's effect size for discovery trait k, that is, the effect of having one more copy of the reference allele at SNP_{kj}. g_{kji} is individual i's genotype at marker j for discovery trait k, coded as having 0,1, or 2 copies of the reference allele at $marker_{kj}$. Conventionally, the $\hat{\beta}_{kj}$ for SNP_j is simply the GWAS k estimate for SNP_{jk}. However, due to local linkage disequilibrium (LD) (i.e. correlation) between SNPs, $\hat{\beta}_{kj}$ captures any effects of the SNP_{kj} and its correlates. Therefore, to correct for the multiple counting problem of effectively counting the effects of markers that are in LD with other markers multiple times, conventionally, markers are thinned down via the process of 'clumping' to a set of uncorrelated markers prior to polygenic score creation. In this study, to avoid a reduction in predictive accuracy and loss of information caused by the conventional approach of LD-based marker pruning and applying a P-value threshold to association statistics, we used *LDpred*[10] (version 0.9.09; https://github.com/bvilhjal/ldpred). *LDpred* is a Bayesian approach that infers the posterior mean effect size of each marker by adjusting the effect size from the discovery GWAS using a prior on effect size and information on the LD between the SNPs from a

reference panel to obtain a posterior estimate of the causal effect for SNP_{jk} independent of the effects of other SNPs. Hence, the *LDpred* GPS for individual *i* for GWAS *k* is the sum of *i*'s genotypes across all SNPs used in the analyses, weighted by the *LDpred* estimates of the genotype effects. The score represents an unbiased estimate of the true genetic burden for individual *i* for trait *k*[10], albeit with low precision.

As recommended by the *LDpred* developers[10], we used the target sample genotype data as the LD reference panel. *LDpred* models a prior probability for the fraction of markers assumed to be causal using a Gaussian mixture weight. We created *LDpred* scores for the following prior probabilities of fraction of causal markers: 0.01, 0.1,1.0.

To account for population stratification, we adjusted the polygenic predictors by the first 10 principal components generated from genotype data prior to the analyses. We used the top 10 PCs as well as genotyping array and plate to create a N*P matrix Z of eigenvectors across the P selected principal components. We then regressed the genetic polygenic predictor onto the eigenvectors as $S = \mu + Z\beta + e$, where μ is the mean and β is a $P\times 1$vector of the regression coefficients, and e is the residual error.

Measures of general cognitive ability (g)

General cognitive ability ('*g*'; intelligence) was assessed in TEDS at ages 7, 9, 10, 12, 14, and 16. For the present analyses we created a longitudinal composite measure of 'g' as a mean of these six assessments. At age 7, 'g' was calculated as a mean of conceptual grouping[11], a WISC similarities test[12], a WISC vocabulary test[12], and a WISC picture completion test[12] all collected over telephone testing. At age 9, 'g' was calculated as a mean of a shapes test[13], a WISC vocabulary test[14], a WISC general knowledge task[14], and a puzzle test[13] ; all tests were administered in booklets sent to the twins by post. At age 10, 'g' was calculated as a mean of the Ravens Standard Progressive Matrices[15], a WISC vocabulary[14], WISC picture completion[12], and a WISC general knowledge test[14]; at age 10

and subsequent assessments, all ‘g’ data were obtained by internet testing. At age 12, ‘g’ was calculated as a mean of the Ravens Progressive Matrices[15], a WISC picture completion[12], a WISC vocabulary[14], and a WISC general knowledge test[14.] At age 14, ‘g’ was calculated as a mean of the Raven’s Progressive Matrices[15] and a WISC vocabulary[14]. At age 16, ‘g’ was calculated as a mean of Mill Hill Vocabulary test[16] and Raven’s Progressive Matrces[15].

References

1 Chang CC, Chow CC, Tellier LC, Vattikuti S, Purcell SM, Lee JJ. Second-generation PLINK: rising to the challenge of larger and richer datasets. *Gigascience* 2015; **4**: 7.

2 Purcell S, Neale B, Todd-Brown K, Thomas L, Ferreira MAR, Bender D *et al.* PLINK: A Tool Set for Whole-Genome Association and Population-Based Linkage Analyses. *Am J Hum Genet* 2007; **81**: 559–575.

3 *R: A Language and Environment for Statistical Computing*. R Foundation for Statistical Computing: Vienna, Austria, 2016.

4 Danecek P, Auton A, Abecasis G, Albers CA, Banks E, DePristo MA *et al.* The variant call format and VCFtools. *Bioinformatics* 2011; **27**: 2156–2158.

5 McCarthy S, Das S, Kretzschmar W, Durbin R, Abecasis G, Marchini J. A reference panel of 64,976 haplotypes for genotype imputation. 2015 doi:10.1101/035170.

6 Howie B, Fuchsberger C, Stephens M, Marchini J, Abecasis GR. Fast and accurate genotype imputation in genome-wide association studies through pre-phasing. *Nat Genet* 2012; **44**: 955–959.

7 Fuchsberger C, Abecasis GR, Hinds DA. Minimac2: Faster genotype imputation. *Bioinformatics* 2015; **31**: 782–784.

8 Consortium TIH 3. Integrating common and rare genetic variation in diverse human populations. *Nature* 2010; **467**: 52–58.

9 Okbay A, Beauchamp JP, Fontana M, Lee JJ, Pers T., Rietveld CA *et al.* Genome-wide association study identifies 74 loci associated with educational attainment. *Nature* 2016; **533**: 539–542.

10 Vilhjálmsson BJ, Yang J, Finucane HK, Gusev A, Lindström S, Ripke S *et al.* Modeling linkage disequilibrium increases accuracy of polygenic risk scores. *Am J Hum Genet* 2015; **97**: 576–592.

11 McCarthy D. *McCarthy Scales of Children’s Abilities*. New York: The Psychological Corporation., 1972.

12 Wechsler D. *Wechsler Intelligence Scale for Children (3rd Ed. UK)*. The Psychological Corporation, 1992.

13 Smith P, Fernandes C, Strand S. *Cognitive Abilities Test 3 (CAT3)*. Windsor: nferNELSON., 2001.

14 Kaplan E, Fein D, Kramer J, Delis D, Morris R. *WISC-III As a Process Instrument (WISC-III-PI)*. New York: The Psychological Corporation., 1999.

15 Raven J, Raven JC, Court J. *Manual for Raven’s Progressive Matrices and Vocabulary Scales*. Oxford: Oxford University Press, 1996.

16 Raven JC, Raven J, Court JH. *The Mill Hill Vocabulary Scale*. Oxford:OPP, 1998.
